# An Africa-wide genomic evolution of insecticide resistance in the malaria vector *Anopheles funestus* involves selective sweeps, copy number variations, gene conversion and transposons

**DOI:** 10.1371/journal.pgen.1008822

**Published:** 2020-06-04

**Authors:** Gareth D. Weedall, Jacob M. Riveron, Jack Hearn, Helen Irving, Colince Kamdem, Caroline Fouet, Bradley J. White, Charles S. Wondji

**Affiliations:** 1 Vector Biology Department, Liverpool School of Tropical Medicine (LSTM), Pembroke Place, Liverpool, United Kingdom; 2 School of Biological and Environmental Sciences, Liverpool John Moores University, Liverpool, United Kingdom; 3 Centre for Research in Infectious Diseases (CRID), Yaoundé, Cameroon; 4 LSTM Research Unit at CRID, Yaoundé, Cameroon; 5 Department of Entomology, University of California, Riverside, California, United States of America; 6 Verily Life Sciences, South San Francisco, California, United States of America; University of Exeter, UNITED KINGDOM

## Abstract

Insecticide resistance in malaria vectors threatens to reverse recent gains in malaria control. Deciphering patterns of gene flow and resistance evolution in malaria vectors is crucial to improving control strategies and preventing malaria resurgence. A genome-wide survey of *Anopheles funestus* genetic diversity Africa-wide revealed evidences of a major division between southern Africa and elsewhere, associated with different population histories. Three genomic regions exhibited strong signatures of selective sweeps, each spanning major resistance loci (*CYP6P9a/b*, *GSTe2* and *CYP9K1*). However, a sharp regional contrast was observed between populations correlating with gene flow barriers. Signatures of complex molecular evolution of resistance were detected with evidence of copy number variation, transposon insertion and a gene conversion between *CYP6P9a*/*b* paralog genes. Temporal analyses of samples before and after bed net scale up suggest that these genomic changes are driven by this control intervention. Multiple independent selective sweeps at the same locus in different parts of Africa suggests that local evolution of resistance in malaria vectors may be a greater threat than trans-regional spread of resistance haplotypes.

## Author summary

Malaria control currently relies heavily on insecticide-based vector control interventions. Unfortunately, resistance to insecticides is threatening their continued effectiveness. Metabolic resistance has the greatest operational significance, yet it remains unclear how mosquito populations evolutionarily respond to the massive selection pressure from control interventions including insecticide-treated nets. Deciphering patterns of gene flow between populations of major malaria vectors such as *Anopheles funestus* and elucidating genomic signature of resistance evolution are crucial for designing resistance management strategies and preventing malaria resurgence. Here, we performed a genome-wide survey of *An*. *funestus* genetic diversity from across its continental range using reduced-genome representation (ddRADseq) and whole genome (PoolSeq) approaches revealing evidence of significant barriers to gene flow impacting the spread of insecticide resistance alleles. This study detected signatures of strong selective sweeps occurring in genomic regions controlling cytochrome P450-based and glutathione s-transferase metabolic resistance to insecticides in this species. Fine-scale analysis of the major pyrethroid resistance-associated genomic regions revealed complex molecular evolution with evidence of copy number variation, transposon insertion and gene conversion highlighting the risk that if this level of selection and spread of resistance continues unabated, our ability to control malaria with current interventions will be compromised.

## Introduction

Insecticide-based mosquito control has been hugely successful in reducing malaria globally [1] but has driven the evolution of insecticide resistance. This presents a serious challenge for malaria control and elimination [2], and may be linked to recent stalling of progress on malaria reduction [3]. Large-scale insecticide use creates strong selective pressures on mosquito populations to evolve resistance. This is made more likely by large effective population sizes and high levels of standing genetic diversity in mosquito populations [4]. Evidence of such evolution can be seen in the patterns of genetic diversity of mosquito populations, with selective sweeps reducing genetic diversity around loci associated with resistance [4–6]. Population genomics can help in identifying molecular mechanisms underlying resistance and in understanding and/or predicting their spread from one population to another given the population structure of the species across its range. *Anopheles funestus* is a major malaria vector throughout sub-Saharan Africa [7]. Resistance to major insecticides is increasingly reported in *An*. *funestus* Africa-wide [8–12]. However, patterns of resistance and underlying resistance mechanisms vary significantly between African regions [6, 12, 13]. It remains to establish whether this is the result of different local selection pressures or the presence of strong barriers to gene flow between populations. Therefore, understanding the mechanisms of resistance and their potential to spread is a priority.

A recent selective sweep spanning a cluster of cytochrome P450 monooxygenase genes in southern Malawi has likely been driven by increased use of insecticide-treated bednets [5]. Reduced genetic diversity was also seen at this locus in other parts of Africa [5]. However, signatures of selection in other regions where other mechanisms of resistance are at play remain uncharacterised. There is therefore a need to expand the analysis of selective sweeps to multiple countries across the range of *An*. *funestus*.

Understanding the population structure of *Anopheles funestus* across sub-Saharan Africa could help predict the spread of new resistance-associated mutations. Previous population genetic analyses, employing a range of molecular markers including microsatellites [14–16], ribosomal DNA [17], mitochondrial DNA [15] and chromosomal inversions [18], indicate at least one major subdivision separating “western” and “eastern” African populations of *An*. *funestus* and a putative “central” population [15]. Genetic divergence among populations is associated with physical geographical barriers, such as the Great Rift Valley [15, 19]. Otherwise, across large areas of the continent, the population structure is relatively “shallow” (*i*.*e*. there is little differentiation), a pattern consistent with recent population expansion [15]. This apparent genetic structure of *An*. *funestus* is also shown through the distribution of recently detected markers of insecticide resistance in this species with resistance alleles for RDL (resistance to dieldrin) [20] and for the glutathione-S transferase epsilon 2 (L119F-GSTe2) [21] only found in West/Central and part of East Africa but completely absent from southern Africa. In contrast, resistance alleles for the cytochrome P450 genes CYP6P9a_R [6] and CYP6P9b_R [22] as well as for the N485I Ace-1 conferring carbamate resistance [23] are all found only in southern Africa and part of East Africa but completely absent from other regions. In this, it is similar to *Anopheles gambiae*, which also shows a broad division between “eastern” and “north-western” African populations and an otherwise shallow population structure [24]. Deciphering the continental patterns of gene flow in this species with novel approaches including genome-wide sequencing of loci is needed to inform and predict the future spread of these resistance markers and other genes of interest.

In this study, we carried out large-scale genomic sequencing of *Anopheles funestus* mosquitoes collected from sites across its sub-Saharan African range. We analysed population structure using a large set of SNP markers and scanned SNP allele frequencies throughout the genome to identify several signatures of strong recent selection near candidate insecticide resistance loci. Furthermore, detailed analysis of a major resistance locus, the CYP6 cluster on chromosome arm 2R, showed patterns of complex molecular evolution including gene amplification and gene conversion. Our analyses suggested that resistance has arisen independently at the same locus in several populations, with consequences for the way resistance or other genes of interest can spread Africa-wide.

## Results

### Population genomic analyses indicate a major subdivision between southern African and other populations suggesting differing population histories

#### Analysis of population structure using ddRADseq

**Fig 1:**
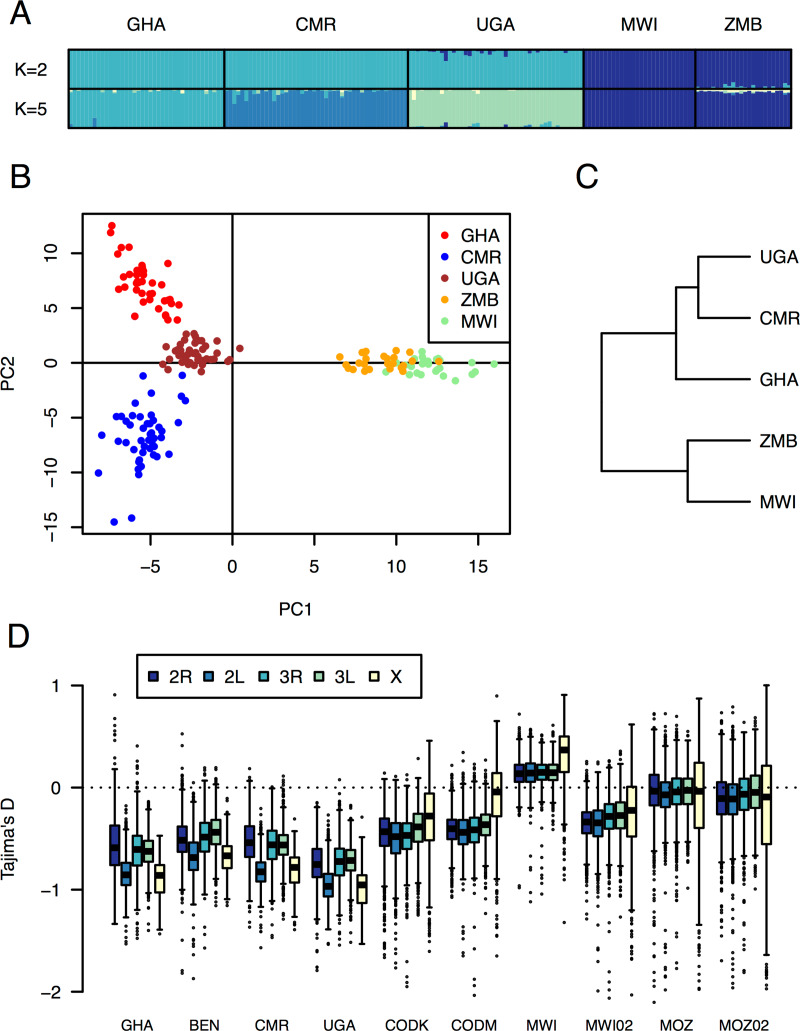
Population structure and history of *An*. *funestus* across its African range. **(**A) STRUCTURE plots showing individual ancestry assignment for ddRADseq genotypes of mosquitoes from 5 sampled populations. Cluster assignment probabilities are shown for each individual (represented by a bar, colours indicate different clusters), for K = 2 clusters (the most likely number, predicted by Evanno’s method) and K = 5 clusters (for the 5 countries sampled). Results show that Zambian and Malawian populations cluster together, even when 5 clusters are specified, indicating they form a single population, diverging from other African populations. (B) Principal component analysis plot showing how individual mosquitoes cluster based on ddRADseq genotypes, showing the separation of Zambia and Malawi from other populations on the first principal component (PC1), and the separation of Ghana, Cameroon and Uganda on the second principal component (PC2). (C) UPGMA dendrogram representing the relative relationships of the 5 populations based on pairwise *F*_*ST*_ among populations (based on ddRADseq genotypes). Again, Zambia and Malawi cluster together, away from the other populations. (D) Boxplots showing the distribution of Tajima’s D across each chromosome arm of the genome (based on PoolSeq data for 8 contemporary African populations and 2 additional population samples from 2002).

We used double digest RAD sequencing (ddRADseq) to analyse population structure in *An*. *funestus* across its range. A total of 1280 SNP markers (a result of stringent filtering to retain only sites genotyped in every individual in every population) were used to compare 5 populations from West [Ghana (Obuasi); n = 39], Central [Cameroon (Mibellon); n = 46], East [Uganda (Tororo); n = 44] and Southern Africa [Malawi (Chikwawa); n = 28 and Zambia (Kaoma); n = 24]. Clear divergence was seen between southern Africa and elsewhere (Fig 1). In STRUCTURE analyses, the most likely number of clusters (K) was 2 (Malawi and Zambia in one, Ghana, Cameroon and Uganda in the other) and even for K>5 (the number of populations sampled) Malawi and Zambia were still assigned to one cluster, while Ghana, Cameroon and Uganda were each assigned to their own (Fig 1A). Principal component analysis showed the same pattern, with the major axis separating the southern populations and the rest and the second axis separating Ghanaian, Cameroonian and Ugandan populations (Fig 1B), as did *F*_*ST*_ across all 1280 markers, which also clearly separated southern African from other populations (Fig 1C). Overall, *F*_*ST*_ ranged from the 0.035, between Cameroon and Uganda, to 0.136 between Malawi and Ghana. The low level of divergence between Cameroon and Uganda, lower than that seen between the geographically closer Cameroon and Ghana, was consistent with previous observations based on microsatellites and individual, insecticide resistance-associated genes such as cytochrome P450s *CYP6P9a* and *b* [5, 6].

#### Analysis of population structure and history using PoolSeq

To extend the analysis of population structure and history to include more populations and more variant sites, we carried out pooled-template whole genome sequencing (PoolSeq) of 40 field collected female mosquitoes (except for Mikalayi in eastern DRC, where 29 mosquitoes were pooled) for 8 populations from West (Ghana and Benin), Central (Cameroon, western DRC and eastern DRC), East (Uganda) and Southern Africa (Malawi and Mozambique).

Sequence data obtained for each pool was trimmed to remove sequencing adapter and low-quality regions and filtered to remove short and unpaired reads (S1 Table) then aligned to the FUMOZ reference genome assembly. Alignments were filtered to remove duplicates and reads with low mapping quality (S2 Table). All variant sites were identified and filtered to remove SNPs at the extremes of coverage depth, before variant calling was carried out (S3 Table). Between 2,096,950 and 4,681,505 SNPs (mean 3,978,975) were identified in each population sample (S3 Table). As each sample was a pool of genomes, allele frequency was estimated at each SNP site and used to analyse population history and to identify genomic regions under selection (described in a later section). We scanned the SNP allele frequencies and estimated Tajima’s D for 50 kb sliding windows throughout the genome. The distributions of Tajima’s D calculated for all windows assigned to each chromosome arm (2L, 2R, 3L, 3R and X) were determined for each population sample (Fig 1D). The X chromosome showed more variable estimates of Tajima’s D than the autosomes, more notably in southern African populations and probably due to lower overall levels of genetic diversity in the X chromosome. Over all chromosomes, the most striking difference was between southern and other populations. Median Tajima’s D values were negative for all populations but were closer to 0 in southern African populations (Malawi and Mozambique) than in populations from further north; two populations from southern DRC showed intermediate levels of D (Fig 1D). Genome-wide negative Tajima’s D suggests historical population expansion in northern and western Africa, with populations in southern Africa nearer to equilibrium. This is consistent with data from *An*. *gambiae*, where “western” populations show evidence of population expansion that “eastern” populations do not [24], and may indicate historical range expansion of the species from a southern African origin, or reflect host demography and land use changes. However, from a practical perspective there appears to be no overall lack of standing genetic diversity (on which selection can act) in the expanding non-southern populations compared to the southern populations (S3 Table).

**Fig 2:**
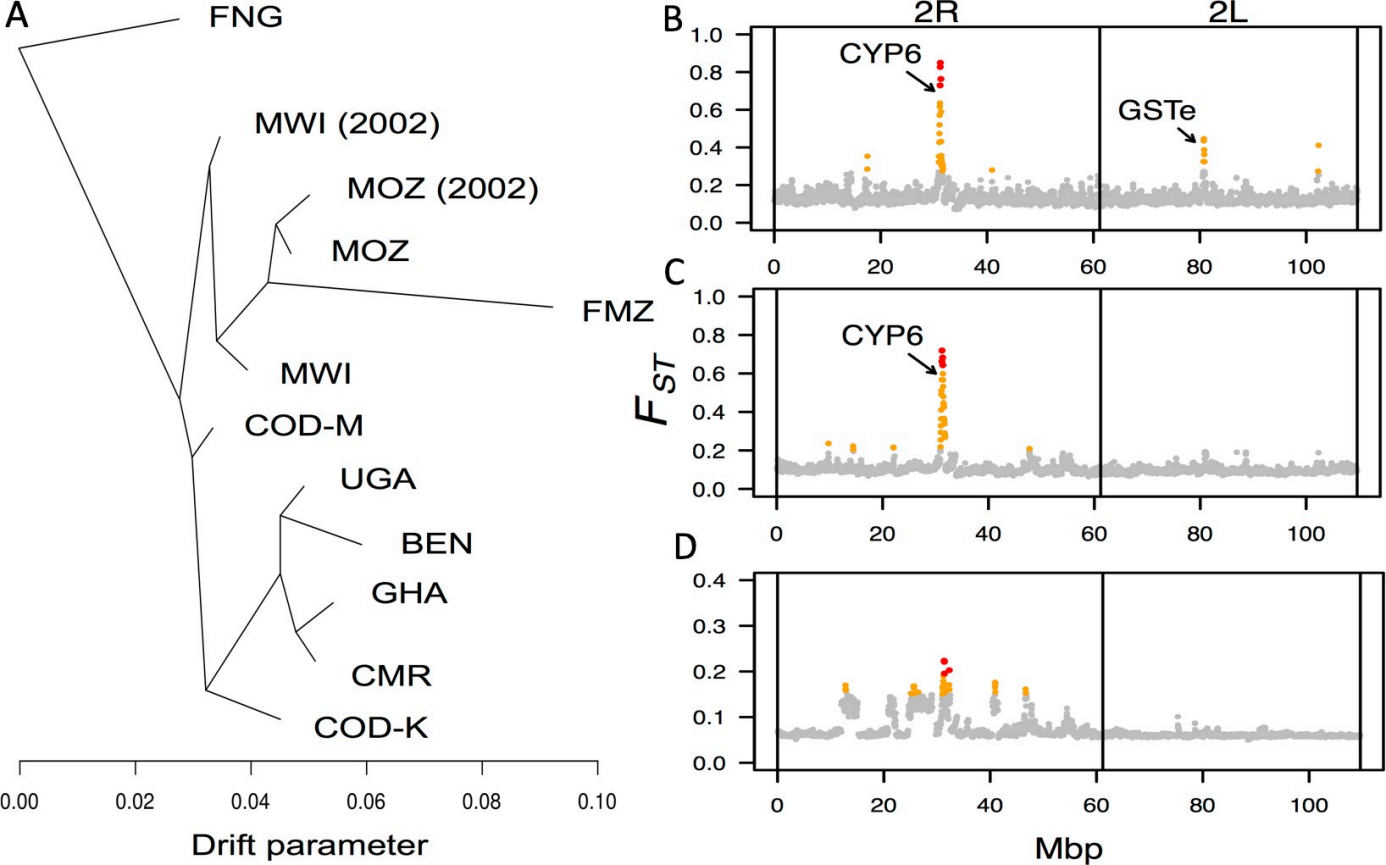
Genetic differentiation between *Anopheles funestus* populations. (A) TreeMix phylogeny showing the relationship between all *An*. *funestus* populations (using PoolSeq data). The drift parameter on the x-axis reflects the amount of genetic drift among populations. The tree was created from all genome-wide SNPs passing filters, without migration edges inferred, and ten iterations of TreeMix produced identical topologies. (B) Genetic differentiation (fixation index *F*_*ST*_) on chromosome 2 between *An*. *funestus* populations from Benin and Mozambique. Each point represents *F*_*ST*_ calculated for a sliding window of 50 kb, moving in 25 kb steps (using PoolSeq data). The highest 1% and 0.1% of *F*_*ST*_ values are shown in orange and red, respectively. Peaks of genetic differentiation at the CYP6 cluster (under selection in both populations) and the GSTe cluster (under selection in Benin only) are indicated. (C) Genetic differentiation (fixation index *F*_*ST*_) on chromosome 2 between *An*. *funestus* populations from Uganda and the Democratic Republic of Congo (Kinshasa). A peak of genetic differentiation at the CYP6 cluster (under selection in both populations) is indicated. (D) Genetic differentiation (fixation index *F*_*ST*_) on chromosome 2 between *An*. *funestus* populations from Ghana and Cameroon, showing around 7 extended regions of elevated divergence on arm 2R.

To further decipher patterns of genetic differentiation between *An*. *funestus* populations across Africa, we analysed historical relationships among populations using a graph representation that allows population splits and migration events. The TreeMix analysis (Fig 2A) generated a tree using the PoolSeq data which clearly separated Southern and Central/Western African populations, consistent with our ddRADseq-based results and published microsatellite-based results [5]. Two major clusters were detected, one made of populations from West (Ghana and Benin), Central (Cameroon) and East/Central (Uganda) Africa. The Congo population from Kinshasa is also closer to this cluster. The second cluster is made of populations from Malawi and Mozambique including the laboratory resistant strain FUMOZ originally from Mozambique. Interestingly, the Mikalayi population from Central Congo is between the two major clusters but closer to the southern one whereas the FANG susceptible lab strain, originally from southern Angola, is on its own.

### *Anopheles funestus* populations display signatures of multiple selective sweeps associated with insecticide resistance loci

Insecticide exposure creates very strong, recent selective pressures upon mosquito populations. This may leave signatures of positive directional selection in the patterns of polymorphism in the genome (reduced genetic diversity and an excess of rare variants) and help to characterise and detect genomic regions associated with resistance. Hypothesizing that insecticide pressure is the strongest recent selection pressure upon mosquito populations, so that most major selective sweeps will be due to exposure to these insecticides, we scanned our PoolSeq samples to detect such signatures. We used the Tajima’s D values for 50 kb sliding windows across the autosomes (the X chromosome was analysed separately due to the highly variable estimates of Tajima’s D probably due to lower overall levels of genetic diversity) to scan the genome and detected multiple selective sweeps. These were seen in some populations but not in others, and some populations showed multiple selective sweeps at different loci (S1 Fig).

One major selective sweep, on chromosome arm 2R (on scaffold KB669169 of the reference genome assembly) was seen in Malawi, Mozambique, Uganda, DRC (Mikalayi) and Benin (S1 Fig). It was less pronounced in, or absent from, DRC (Kinshasa) and Cameroon, while in Ghana pronounced positive Tajima’s D was seen (considered further in later sections). The sweep spans a major pyrethroid resistance locus previously reported as ‘resistance to pyrethroid 1’ (*rp1*) and sequenced on a 120 kb BAC (accession PRJEB37305) [25]. The locus contains a cluster of cytochrome P450 monooxygenase genes of the CYP6 family (as well as two carboxylesterase genes) and genes in this CYP6 cluster are under selection in *An*. *funestus* and *An*. *gambiae* populations [4, 5, 25].

Another selective sweep, on chromosome arm 2L (scaffold KB669036) and seen only in Benin (S1 Fig), encompassed a cluster of GST epsilon genes associated with DDT and pyrethroid resistance and shown to be under selection in *An*. *gambiae* [4, 21].

In addition to these, additional possible signature of selection were seen on chromosome arm 2L (scaffold KB668697, containing 28 annotated genes) and at the telomeric end of 3R (scaffold KB668905, containing 21 annotated genes) in all populations (S1 Fig). These warrant further study, but here we focused upon the two selective sweeps spanning known mediators of metabolic resistance.

#### Divergence between African regions at loci under selection and putative chromosomal inversions

Pairwise *F*_*ST*_ estimated (using ddRADseq data) across the genome reflected the broad trend (Fig 1A–1C) of substantial divergence between southern African populations and those from other parts of the continent and showed elevated *F*_*ST*_ throughout the genome (S2 Fig). However, in addition to this, divergence was distributed unevenly throughout the genome, with the most divergent regions tending to occur on chromosome arm 2R, followed by 3R, with relatively little on 2L and 3L in comparison. The most extreme divergence coincided with selective sweeps, particularly around the CYP6 gene cluster on 2R in some populations (Fig 2B and 2C) and the GST epsilon gene cluster in Benin. This suggests that different haplotypes are under selection in different populations, consistent with two possible scenarios: the same selected allele in different populations with hitchhiking of different flanking haplotypes due to recombination, or multiple independent selective sweeps on different alleles selected either for resistance to the same insecticides or to different ones. This is explored further in later sections.

In comparisons between Ghana and other populations, extended regions of elevated divergence were seen on chromosome arm 2R (Fig 2D, S2A–S2D Fig). This is consistent with patterns of sequence divergence seen at chromosomal inversions and occurs in the chromosome arm richest in reported inversions [26]. This suggests the sampled Ghanaian population may be enriched for inversions not seen, or at low frequencies, in other populations. Even against this background, a peak of *F*_*ST*_ is seen at the CYP6 gene cluster between Ghana and Cameroon, neither of which displayed signatures of a selective sweeps at this locus (Ghana displaying the opposite signature: positive Tajima’s D). The presence of two independent haplotypes under selection in Ghana, possibly on different inversion backgrounds, might partly explain these patterns.

### Characterisation of the selective sweep and complex molecular evolution of the CYP6 gene cluster on chromosome arm 2R

**Fig 3:**
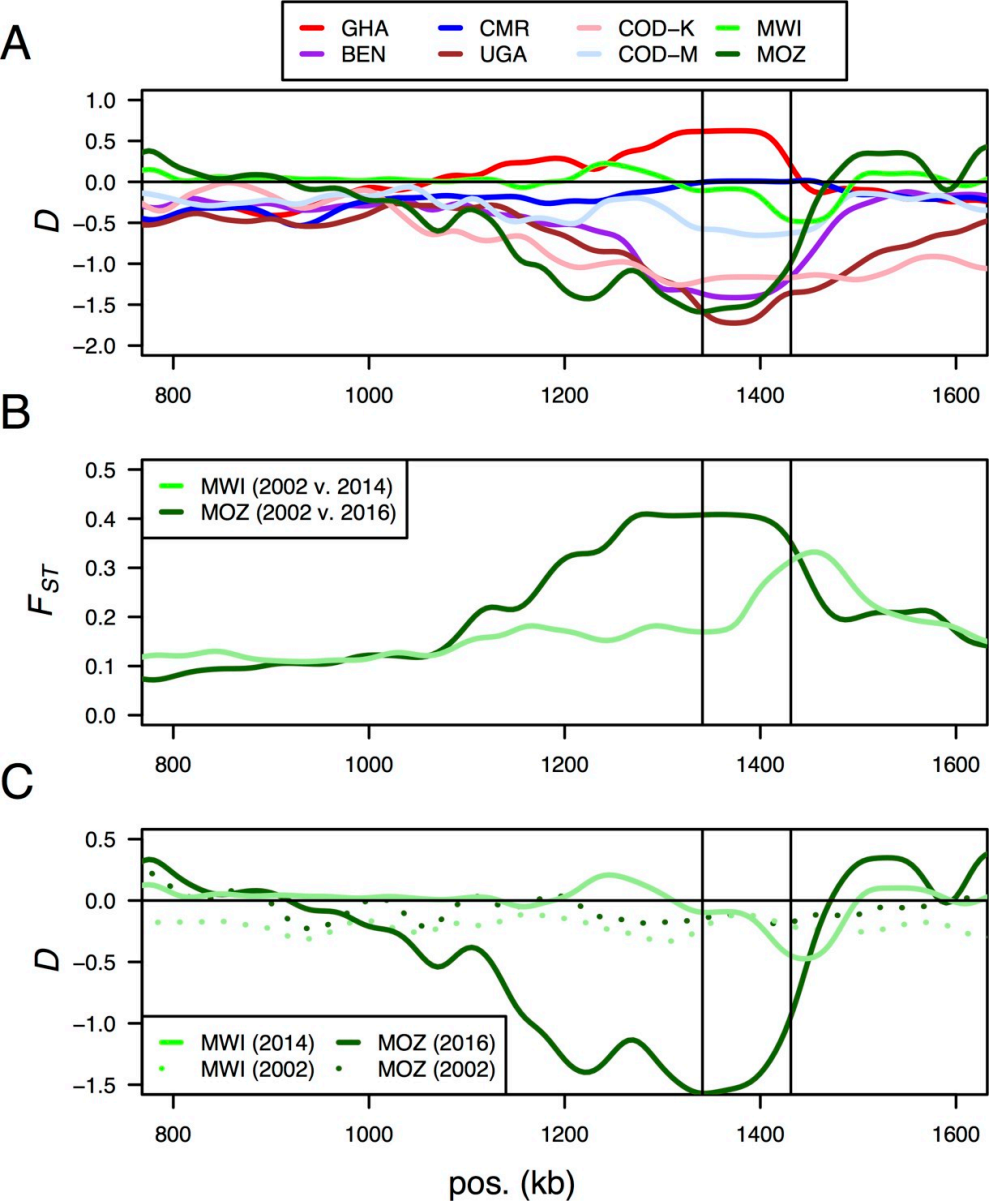
A selective sweep associated with insecticide resistance in *An*. *funestus* populations across Africa on chromosome arm 2R. (A) A selective sweep spanning a CYP6 gene cluster associated with pyrethroid resistance in Benin (BEN), Uganda (UGA), Kinshasa, DRC (COD-K), Mozambique (MOZ) and Malawi (MWI). Lines are kernel-smoothed Tajima’s D values calculated for 10 kb windows moving in steps of 5 kb. The vertical lines in all panels indicate the start and end of the CYP6 cluster. The sweep is less pronounced for Malawi, in part due to the complete fixation of reference alleles, and is clearer in the temporal analyses. (B) Temporal changes in allele frequencies at the CYP6 gene cluster in Malawi (light green) and Mozambique (dark green). Lines show kernel-smoothed *F*_*ST*_ (for a 10 kb sliding window moving in 5 kb steps) between 2002 and 2014/2016 and show elevated *F*_*ST*_ spanning the CYP6 cluster (indicated by the vertical lines). (C) Temporal changes in Tajima’s D, showing that the peaks of high *F*_*ST*_ are due to the loss of diversity (excesses of rare alleles, negative Tajima’s D) in the Malawi 2014 population (solid light green line) and the Mozambique 2016 population (solid dark green line), compared to the 2002 populations (dashed lines) which show no evidence of deviation from selective neutrality. Lines show kernel-smoothed Tajima’s D (for a 10 kb sliding window moving in 5 kb steps). Positions (‘pos’, in kb) refer to scaffold KB669169.

To further characterise the selective sweep and haplotypes of the CYP6 cluster on chromosome arm 2R (Fig 3A), we extended a previous temporal population genomic analysis of the selective sweep in southern Africa [6] and carried out fine scaled analysis of sequence alignments to identify numerous previously unreported cases of complex molecular evolution segregating within the species in different populations.

#### New temporal analysis confirms the recent age of the selective sweep

We previously identified this selective sweep in Malawi and used a temporal analysis of allele frequency changes in the population between 2002 and 2014 to show the very recent age of the sweep [5]. Here, we extended this analysis with additional southern African populations from Mozambique (collected in 2002 before scale-up of insecticide-treated interventions and 2016 post-intervention). We found a similar pattern as we saw in Malawi, though even more striking (Fig 3). In both populations, peaks of high *F*_*ST*_ occurred near the CYP6 gene cluster (Fig 3B), indicating extreme divergence in this region between populations sampled in 2002 (before scale up in the use of insecticide-treated bed nets) and in 2014/2016 (when their use was widespread). Maximum temporal divergence was greater in Mozambique (*F*_*ST*_>0.4) than in Malawi (*F*_*ST*_>0.3) and occurred over a broader region spanning the CYP6 cluster (Fig 3B). Analysis of Tajima’s D over the same genomic region confirmed that the extreme temporal divergence was due to a loss of genetic diversity since 2002 in both populations, though more extreme and over a larger region in Mozambique (Fig 3C). We also previously showed that the selected haplotype in Malawi (in 2014) contained an insertion of approximately 6.5 kb between two paralogous genes: *CYP6P9a* and *CYP6P9b* [6]. The same insertion was found in the Mozambique population (in 2016) and was absent from or at very low frequency in these populations in 2002 (Table 1; encoded as a deletion ‘rp1:37410–43954’ relative to the FUMOZ *rp1* BAC, that contained the insertion). The insertion was fixed in both the 2014 Malawi and 2016 Mozambique populations. It was also seen at approximately 80% frequency in a population from eastern DRC (Mikalayi) in 2015, but absent from western DRC (Kinshasa) in 2015 (Table 1). Taken together with evidence from a linked marker [6], this suggests a relatively recent insertion, possibly in or near Mozambique, followed by rapid selection and spread north and westwards, to eastern DRC and Tanzania [6]. However, while this selected, insertion-containing haplotype is associated with massive over-expression of *CYP6P6a* and *b* genes in southern Africa [6], the same region (without the insertion) is also under selection in other parts of Africa. This is consistent with evidence from the *F*_*ST*_ analyses of divergence among populations suggesting selection on different haplotypes, and possibly causal alleles, in different mosquito populations.

**Table 1:** Structural and copy number polymorphism in the CYP6 gene cluster.

Location/ colony	Year	Pooled genomes	Structural/copy number variant ^1^	Notes
FUMOZ	n/a	38	None	
Ghana (Obuasi)	2014	40	rp1:37410–43954	Deletion (6.5kb) between *CYP6P9a* and *CYP6P9b* (deletion fixed)
			rp1:17910–24836	Tandem duplication (6.9kb) spanning *CYP6AA1* and partial *CYP6AA2*
			rp1:46407–52668	Tandem duplication (6.2kb) spanning *CYP6P5* and *CYP6P4a*
			rp1:10311–71182	Tandem duplication (60.8kb) spanning entire CYP6 cluster
Benin (Kpome)	2015	40	rp1:37410–43954	Deletion (6.5kb) between *CYP6P9a* and *CYP6P9b* (deletion fixed)
			rp1:18643–25686	Tandem duplication (7.0kb) spanning *CYP6AA1* and partial *CYP6AA2*
Cameroon (Mebellon)	2014	40	rp1:37410–43954	Deletion (6.5kb) between *CYP6P9a* and *CYP6P9b* (deletion fixed)
			rp1:19133–25617	Tandem duplication (6.4kb) spanning *CYP6AA1* and partial *CYP6AA2*
			rp1:19043–35875	Tandem duplication (16.8kb) spanning *CYP6AA1*, *CYP6AA2*, 2x carboxylesterases, *CYP6P15P* and partial *CYP6P9a*
Uganda (Tororo)	2014	40	rp1:37410–43954	Deletion (6.5kb) between *CYP6P9a* and *CYP6P9b* (deletion fixed)
DRC (Kinshasa)	2015	40	rp1:37410–43954	Deletion (6.5kb) between *CYP6P9a* and *CYP6P9b* (deletion fixed)
			rp1:46771–50640	Tandem duplication (3.8kb) spanning *CYP6P5*
			rp1:46864–49106	Deletion (2.2kb; within duplicated region) spanning *CYP6P5*
DRC (Mikalayi)	2015	29	rp1:37410–43954	Deletion (6.5kb) between *CYP6P9a* and *CYP6P9b* (approx. 20% deletion, 80% insertion) ^2^
Malawi (Chikwawa)	2014	40	None ^3^	
Malawi (Chikwawa)	2002	40	rp1:37410–43954	Deletion (6.5kb) between *CYP6P9a* and *CYP6P9b* (deletion fixed)
Mozambique (Manhica)	2016	40	None	
Mozambique (Morrumbene)	2002	40	rp1:37410–43954	Deletion (6.5kb) between *CYP6P9a* and *CYP6P9b* (deletion nearly fixed) ^4^

^1^ The labelling system refers to the nucleotide positions of the left and right breakpoints on the rp1 BAC (accession PRJEB37305).

^2^ Approximate quantification of insertion/deletion based on the number of chimeric and non-chimeric reads at each breakpoint.

^3^ A single read supports the deletion variant, all others support the insertion variant.

^4^ A single read supports the insertion variant, all others support the deletion variant.

#### Numerous independent gene duplication events have led to gene copy number polymorphism (CNP) in the CYP6 cluster:

The region spanning the CYP6 cluster in the *An*. *funestus* FUMOZ reference genome assembly (on scaffold KB669169) contains a number of assembly gaps near and within the CYP6 cluster, making very fine-scaled analysis of sequence alignments difficult. To solve this, we re-aligned the PoolSeq data to a 120kb BAC sequence, with no gaps, containing the CYP6 cluster (‘*rp1*’; accession PRJEB37305) [25]. We used these alignments to analyse molecular evolution in the gene cluster and to identify several gene duplications.

**Fig 4:**
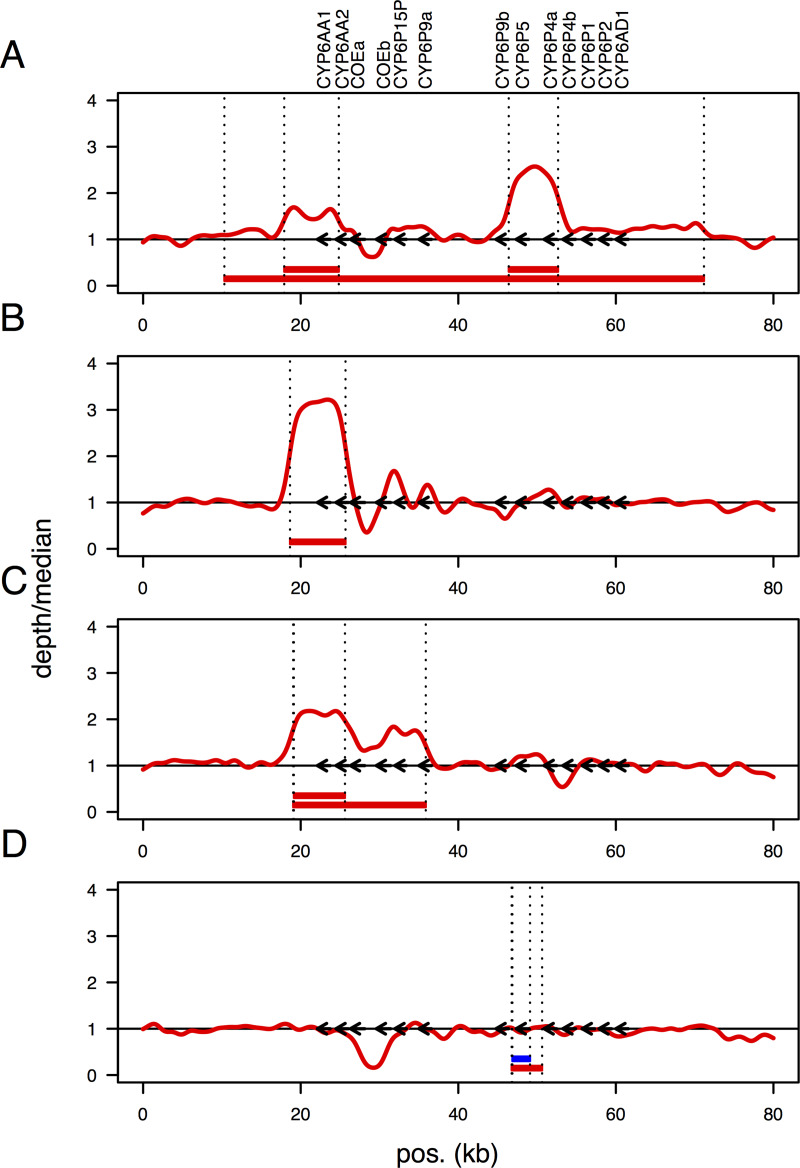
Structural polymorphism in the CYP6 cluster. Coverage depth, relative to the genome-wide median, across the CYP6 cluster for pooled population samples from (A) Obuasi, Ghana, (B) Kpome, Benin, (C) Mebellon, Cameroon and (D) Kinshasa, DRC. Red lines show the kernel-smoothed coverage depths. The horizontal black line indicates coverage depth equal to the genome-wide median. Arrows indicate locations of genes in the CYP6 cluster (labelled at the top). Horizontal red bars indicate tandem duplications confirmed by identification of breakpoints (vertical dotted lines) and the blue bar indicates a deletion. Positions (‘pos’, in kb) refer to the rp1 BAC (PRJEB37305).

**Fig 5:**
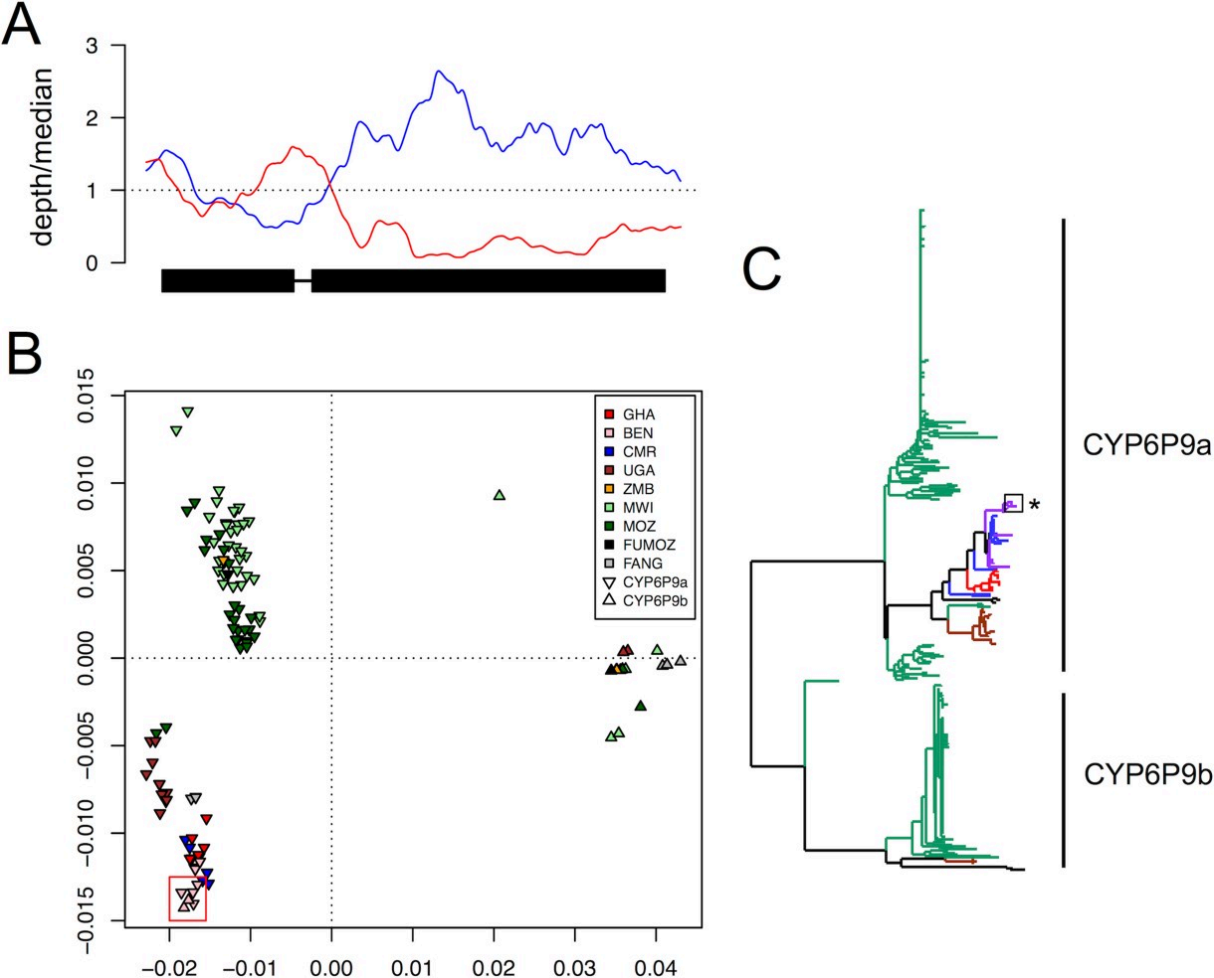
Gene conversion between *CYP6P9a* and *b* in Benin. (A) Coverage depth (relative to the genomic median) across *CYP6P9a* (blue line) and *CYP6P9b* (red line) in the Benin population, suggesting that a large proportion of reads from ‘*CYP6P9b*’ align to *CYP6P9a*. The gene is shown at the bottom (transcribed from right to left), thick black regions indicating exons and the thin black line the intron. (B) Multidimensional scaling (MDS) plot based on pairwise sequence divergence (uncorrected) among 400 *CYP6P9* sequences (281 *CYP6P9a*; 119 *CYP6P9b*). The red box indicates the location of Benin CYP6P9b genes among CYP6P9a genes. The plot also shows the divergence in *CYP6P9a* between southern Africa (Zambia Malawi, Mozambique) and other parts of the continent (Ghana, Benin, Cameroon, Uganda). (C) Neighbour-Joining tree for 281 *CYP6P9a* and 119 *CYP6P9b* sequences. Branches are coloured by geographical region: southern Africa (Malawi, Mozambique, Zambia) in green; Uganda (brown); Ghana (red); Cameroon (blue); Benin (purple). All *CYP6P9b* are clustered together except for 4 sequences from Benin, whose positions among the *CYP6P9a* clade are indicated by a box and an asterisk. Within *CYP6P9a* there are two major clusters, one consisting predominantly of southern African sequences and the other consisting predominantly of sequences from elsewhere in Africa.

The CYP6 gene cluster contains a core set of genes conserved among *Anopheles* species but at least three cases of gene duplication have occurred in the lineage leading to *An*. *funestus* (detectable in both the FUMOZ reference genome assembly and in the FUMOZ *rp1* BAC). These tandem duplications, of *CYP6P3* (so called in *An*. *gambiae*, duplicated to form *CYP6P9a* and *CYP6P9b* in *An*. *funestus*), *CYP6P4* (duplicated to form *CYP6P4a* and *CYP6P4b*) and a carboxylesterase gene, highlight the evolutionary plasticity of the locus. In addition to these among-species differences, we identified complex polymorphisms segregating within the species. As well as the 6.5 kb insertion between *CYP6P9a* and *b* described previously [6] and earlier in this report, we identified several gene copy number polymorphisms (Table 1; Fig 4; S3 Fig), a complex combination of gene duplication and deletion (Table 1; S3E Fig), and gene conversion (Fig 5) within the CYP6 gene cluster.

While West and Central African populations (Benin, Ghana, Cameroon) showed no evidence of the 6.5 kb insertion between *CYP6P9a* and b seen in southern Africa (Table 1), six different duplications were identified (Fig 4A–4C). In Benin, one duplication (‘rp1:18643–25686’, left and right breakpoints occurring at positions 18643 and 25686 on the rp1 BAC) spanned *CYP6AA1* and part of *CYP6AA2*, was at or near fixation in the population and depth of coverage suggested a configuration of 3–4 tandemly arrayed copies (Fig 4B; S3C Fig). In Cameroon, there were two duplications (Fig 4C; S3D Fig). One (rp1:19133–25617), like in Benin, spanned *CYP6AA1* and part of *CYP6AA2* but its breakpoints were at different positions, suggesting a unique duplication event. Another, longer duplication (rp1:19043–35875) spanned all of *CYP6AA1*, *CYP6AA2*, the two carboxylesterase genes, *CYP6P15F* and the 3’ half of *CYP6P9a*. In Ghana, three tandem duplications were identified (Fig 4A; S3A and S3B Fig). One (rp1:17910–24836), like in Benin and Cameroon, spanned *CYP6AA1* and part of *CYP6AA2* but its breakpoints were at different positions again to both of these populations, suggesting yet another unique duplication event. Another (rp1:10311–71182) was 60.8 kb long and contained the entire CYP6 cluster. A third (rp1:46407–52668) spanned *CYP6P5* and *CYP6P4a*. The leftmost breakpoint occurred approximately 30bp upstream of *CYP6P9b* and the rightmost approximately 170bp upstream of *CYP6P4a*. Both genes are significantly over-expressed in this population [6], and increased gene copy number may partly explain this. Given the proximity of the leftmost breakpoint to the start of the *CYP6P9b* gene, at least one copy of *CYP6P4a* would have a portion of the *CYP6P9b* promoter upstream of it and this novel promoter sequence may also contribute to the over-expression of *CYP6P4a*.

Neither Cameroon nor Ghana showed selective sweeps at this locus and both show multiple different duplications. The PoolSeq data did not allow us to estimate copy number (as the alignment was a mixture of genomes) or linkage between the different duplications. Whether the pattern of polymorphism reflects neutral standing genetic diversity at the locus or the presence of multiple selective sweeps overlaid at the same locus could not be determined from the data, though the positive Tajima’s D seen at this locus in the Ghanaian population does suggest two or more haplotypes driven to higher than expected frequencies by selection. Haplotypes containing the whole region in duplicate may also contribute to this signal. Individual whole genome sequencing in this population may resolve this.

In central Africa, the western DRC (Kinshasa) population showed a duplication containing a deletion within it (Fig 4D; S3E Fig). The duplication (rp1:46771–50640) spanned and fully contained *CYP6P5*. The deletion (rp1:46864–49106), nested within this duplication, also spanned and fully contained *CYP6P5*. The result was to effectively replace the upstream promoter of *CYP6P9b* with that of *CYP6P5* which may have profound effects upon the expression of *CYP6P9b* in this population (though we did not have data to test this here). By contrast, the eastern DRC (Mikalayi) population showed no evidence of this duplication, only the 6.5 kb insertion between *CYP6P9a* and *b* seen in southern African populations (which also showed no evidence of duplications, only the 6.5 kb insertion).

In the population from Tororo, Uganda despite evidence of a major selective sweep spanning the CYP6 gene cluster no duplications were seen and there was no evidence for the 6.5 kb insertion between *CYP6P9a* and *b*. This suggests that an allele not associated with a CNP or other forms of complex molecular evolution are under selection in this population.

#### Gene conversion has occurred between the paralogous *CYP6P9a* and *CYP6P9b* genes in Benin

In the population from Kpome, Benin, an unusual pattern of sequence coverage at the CYP6P9a and b genes prompted us to investigate further. In this sample, unlike in other samples, coverage was higher than expected in the first exon of *CYP6P9a* and lower in *CYP6P9b* (Fig 5A). We hypothesized that this pattern was due to gene conversion: a non-homologous recombinational process by which the sequence of one gene is transferred to another gene by its use as a template during DNA repair. The relatively deeper coverage of *CYP6P9a* suggests conversion of the *CYP6P9b* gene to make it *CYP6P9a*-like. Analysis of 400 published *CYP6P9a* (n = 281) and *CYP6P9b* (n = 119) gene sequences supported our hypothesis. A multidimensional scaling (MDS) plot based on pairwise sequence divergence among the genes showed the Benin *CYP6P9b* genes located among *CYP6P9a* genes (Fig 5B). A phylogeny showed the same: *CYP6P9a* and *CYP6P9b* cluster separately (100% bootstrap support), separated by a long branch but the Benin *CYP6P9b* gene sits within the *CYP6P9a* cluster (other than these, only one Malawi *CYP6P9b* gene fell outside of the main *CYP6P9b* cluster, but not within the *CYP6P9a* cluster) (Fig 5C). As both genes are implicated in pyrethroid resistance and efficiency of metabolism is allele-specific [27], such gene conversion could have a major impact on resistance in Benin, potentially effectively doubling the amount of *CYP6P9a* in affected mosquitoes. It is also important to identify cases of gene conversion as they may affect the ability to accurately estimate gene expression levels due to mis-assignment of RNAseq reads or microarray signal and possibly also incorrect primer binding and amplification in qPCR.

### Characterisation of the selective sweep and selected haplotype spanning a cluster of glutathione S-transferase epsilon genes associated with insecticide resistance on chromosome arm 2L

**Fig 6:**
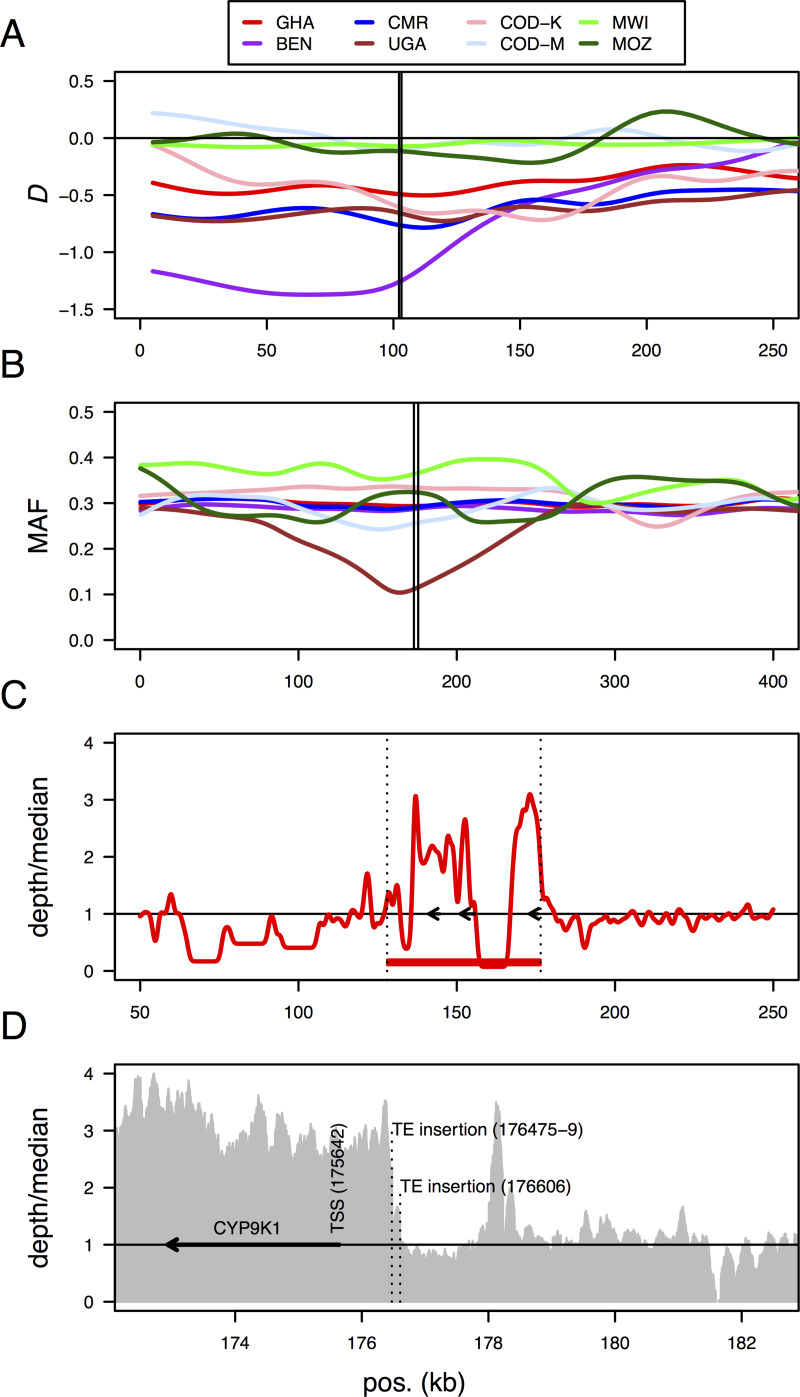
Signatures of selection spanning other resistance associated genomic loci. A) A selective sweep spanning a Glutathione S-transferase (GST) epsilon gene cluster associated with DDT and pyrethroid resistance in Benin (BEN). The gene cluster contains *GSTe2*, which is highly over-expressed in Benin. Lines are kernel-smoothed Tajima’s D values calculated for 10 kb windows moving in steps of 5 kb. The vertical lines indicate the start and end of the *GSTe* cluster. (B) Loss of genetic diversity (reduced minor allele frequencies, MAF) spanning a highly over-expressed *CYP9K1* P450 gene on the X chromosome in Uganda. Lines are kernel-smoothed minor allele frequencies (MAF) as these showed the sweep more clearly that Tajima’s D. The vertical lines indicate the start and end of the *CYP9K1* gene. (C) Increased copy number in the Uganda population of a region of the X chromosome containing three genes (arrows), from left to right: AFUN007547, AFUN007548 and AFUN007549 (*CYP9K1*). The red line indicates coverage depth relative the genomic median, with a horizontal black line at 1. The red rectangle and vertical dotted lines indicate the putative duplicated region. (D) One edge of the region of increased coverage depth (the grey plot) on the X chromosome in the Uganda population, upstream of the *CYP9K1* gene (black arrow), corresponds to a putative transposable element insertion in the genomes of mosquitoes in Tororo, Uganda.

In addition to the CYP6 cluster sweep on 2R, the *An*. *funestus* population from Kpome, Benin also showed evidence of a selective sweep on chromosome arm 2L (Fig 6A), spanning a cluster of GST epsilon genes containing *GSTe2* (AFUN015809), an allele of which (GSTe2-L119F) is associated with DDT and pyrethroid resistance [21]. The population is highly resistant to pyrethroids, DDT and carbamates [9], the resistant TTT (Phenylalanine) allele of GSTe2-L119F is close to fixation and GSTe2 is also over-expressed [6, 13]. The selected haplotype in Benin was analysed and showed a number of differences compared to FUMOZ (S4 Fig). Two regions of low coverage were seen, upstream of *GSTe3* at one end of the cluster and between *GSTe6* and *GSTe8* at the other end (S4A Fig). These could indicate deletions or structural variations, though no further evidence from the alignment supported the latter. Over-expression of *GSTe2* could be linked to a transposon insertion upstream of the transcription start site (TSS) of *GSTe2*, in the 3’ un-translated region of *GSTe1* (S4B Fig), or with a 1 bp deletion (G) immediately adjacent to the TSS of *GSTe2* (S4C Fig). Elsewhere in the gene cluster, *GSTe6* (AFUN016008) shows evidence of partial tandem duplication (S4E Fig), though the duplication breakpoints fall within the gene. Whether this would make *GSTe6* non-functional or create a functional chimeric gene could not be determined. The alignment confirmed the near fixation (98%) of the resistant TTT (F) allele of GSTe2-L119F reported previously (S4D Fig) [9, 21]. By contrast, the Ghanaian and Cameroonian samples showed no strong evidence of a selective sweep at the GST epsilon cluster (Fig 6A) and the GSTe2-L119F TTT (F) allele was present at much lower frequencies (31% in Ghana, 27% in Cameroon; S4D Fig). In Ghana and Cameroon, GSTe2 is over-expressed [6], but to a lesser extent than in Benin. Taken together with the genetic evidence, this might suggest admixed populations in the sampled Ghana and Cameroon populations, or overlapping partial or soft selective sweeps (consistent with the evidence from the CYP6 cluster in Ghana). However, the real scenario is difficult to discern from the pooled data as where genotypes are mixed (in the absence of a single fixed, selected haplotype) haplotype information is lost.

### A selective sweep on the X chromosome in Uganda is associated with a copy number amplification and over-expression of the *CYP9K1* gene

Over-expression of *CYP9K1* has been associated with deltamethrin resistance in *An*. *coluzzii* [28]. *CYP9K1* is overexpressed in Ugandan *An*. *funestus* [6, 13]. We found evidence of a selective sweep spanning *CYP9K1* on the X chromosome (scaffold KB668367) in Uganda not found in the other populations (Fig 6B, S5 and S6 Figs). This sweep coincides with over-expression of the gene in Uganda but not in other populations (S5 Fig, [6, 13]). Detailed inspection of the Uganda PoolSeq alignment also indicated that a region approximately between positions 128,000 to 176,500 on the scaffold (coincident with the selective sweep and containing *CYP9K1* and two other genes) was duplicated, based on increased coverage depth in Uganda (Fig 6C, S6 Fig) that was not seen in other populations. Exact breakpoints flanking the duplicated region (defined by chimeric reads spanning putative breakpoints) could not be identified, possibly due to the poor quality of the reference assembly in this region (the scaffold contains many assembly gaps). At the rightmost edge of the putative duplication, approximately 830 bp upstream of the transcription start site of *CYP9K1*, the aligned data suggested the presence of a transposon in the Ugandan PoolSeq data that was absent from the reference genome (Fig 6D, S6 Fig). This evidence consisted of a putative target site duplication (GAAATTTG at KB668367:176471–176478) and terminal inverted repeats (“GGC TAA AGA GTA GAA AGA GCC CAC AAG TT” or the longer “GCT AAA GAG TAG AAA GAG CCC ACA AGT TGG TAG GTG GTT CCC AGG TTG GAC CAT TGC TCC GCC ATA TTG AAA GT”) seen in the clipped PoolSeq reads but not in the reference sequence, indicating the presence of a mariner-type element (inferred by BLAST similarity of the clipped reads to known mariner elements), found at multiple other locations in the FUMOZ reference genome but not at this region (S6 Fig). Taken together the evidence suggests gene amplification of *CYP9K1* in Uganda, possibly a result of transposon-driven genomic instability, has caused over-expression in this population [6, 13] and that this has been under selection, possibly as it confers resistance to deltamethrin [6, 13, 28]. These results are consistent with previous reports of directional selection on highly over-expressed resistance genes [29].

## Discussion

Analysis of genome-wide genetic diversity of *Anopheles funestus* across its continental range showed evidence of population structure and identified signatures of recent selection from insecticide use helping to predict patterns of spread of major resistance loci in this major malaria vector in Africa.

### *An*. *funestus* populations exhibit strong genetic structure across Africa

We found population subdivision between southern Africa and elsewhere on the continent. *An*. *funestus* populations from western Zambia and southern Malawi appear to be a single contiguous population, whereas both are significantly divergent from populations from West, Central and East Africa (Ghana, Cameroon and Uganda). This barrier to gene flow is similar to previous genetic differentiation patterns obtained from microsatellite markers [5, 15]. Recent profiles of the geographical distribution of key insecticide resistance alleles in this species with markers have also supported this subdivision with markers such as L119F-GSTe2 [21] and the A296S-RDL predominant in West/Central Africa but absent in southern [20] and the metabolic resistance markers CYP6P9a_R [6] in contrast predominant in southern but absent elsewhere. The Ugandan population, from Tororo, is west of the Great Rift Valley, which has been suggested as a geographical barrier preventing gene flow across it for *An*. *funestus* [19] and *An*. *gambiae* [30]. Our data are consistent with this hypothesis. Understanding the continent-wide population structure of the species is important for understanding the spread of resistance mutations between African regions. A major resistance-associated haplotype in southern Africa at a cluster of CYP6 genes appears to be spreading north and west (possibly from an origin in Mozambique). A large (6.5 kb) insertion in this haplotype between two paralogous pyrethroid-metabolising genes (*CYP6P9a* and *b*) is detectable in Mozambique and Malawi, where it is at or near to fixation. It is also detected at intermediate frequencies in Mikalayi in south-eastern DRC (but not in Kinshasa in western DRC) and in Tanzania. Our data suggest that it may be prevented from spreading across the rift valley in east Africa, but how it will spread across central Africa is unknown.

Our observation of multiple, independent selective sweeps at the CYP6 locus in different regions of Africa suggests that resistance is associated with different haplotypes in different regions of Africa. There is a risk that resistance haplotypes could spread among regions and even combine to create multiple- and super-resistant mosquitoes. Therefore, it is important to understand the factors defining population structure of *An*. *funestus* across its range. Currently, the relative contributions of isolation by geographical distance, the presence of physical geographical barriers such as the Rift Valley and genomic barriers to recombination, such as chromosomal inversions, are not clearly defined. Each of these has different consequences for the evolution of resistance. For instance, if populations are simply separated by geographical distance then one may expect resistance haplotypes to spread over time and to mix, creating multiple- and super-resistant mosquitoes that will seriously jeopardise future vector control. Conversely, barriers to gene flow, whether geographical (e.g rift valley) [30] or evolutionary adaptive factors (chromosomal inversions) [31], may more effectively limit the continental spread of resistance haplotypes. However, autochthonous selection of resistance haplotypes in different regions makes it more difficult to develop molecular markers for monitoring resistance across Africa and complicates the application of novel malaria control approaches such as gene drives or symbiont-based control that rely on the natural spread through a population of the genotype or symbiont.

A previous study of the population genetics of *An*. *funestus* across its range reported signatures of population expansion in the Western part of its range [15]. Our whole-genome data support this, with sampled populations from Ghana, Benin, Cameroon, Uganda and DRC showing negative values of Tajima’s D throughout the genome, indicative of population expansion. Populations from Malawi and Mozambique showed genome-wide Tajima’s D values closer to equilibrium. Similar patterns are seen in other major malaria vector species, *Anopheles gambiae* and *Anopheles coluzzii*, where large-scale whole genome sequencing suggests their populations have expanded north of the Congo basin and west of the East African Rift [4].

### Contrasting signatures of selection across African populations of *An*. *funestus* correlate with restrictions to gene flow

Three genomic regions associated with insecticide resistance exhibited signatures of strong recent positive selection in different populations. These were: a cluster of CYP6 cytochrome P450 monooxygenase genes on chromosome arm 2R; a cluster of Glutathione S-Transferase epsilon genes on chromosome arm 2L; and a region containing the *CYP9K1* gene on the X chromosome. Regional differences were observed between populations, in line with patterns of gene flow observed with ddRADseq.

Some selective sweeps were seen only in some populations: one associated with *CYP9K1* only in an East African population (Uganda) and one associated with a GST epsilon gene cluster containing *GSTe2* only in a West African population (Benin). Signatures of selection spanning a cluster of CYP6 cytochrome P450 monooxygenase genes on chromosome arm 2R were more geographically widespread, yet showed patterns specific to different regions. Extreme differentiation among populations (seen as high *F*_*ST*_) at this locus may indicate the selection of different haplotypes in different populations. However, the same signature could also result from selection on the same mutation in all populations, with hitchhiking of different genetic backgrounds flanking this mutation resulting from recombination unique to each population. We suggest that, while the latter scenario is clearly occurring, different haplotypes are being selected for in different populations. For example, while the shape and extent of the selective sweeps differ between neighbouring Mozambique and Malawi, both contain a large insertion between *CYP6P9a* and *CYP6P9b* in the swept haplotype and both populations over-express these genes, massively so for *CYP6P9a* [6]. By contrast, this insertion is absent from other populations that nevertheless show evidence of selective sweeps at this locus, such as Benin and Uganda, where *CYP6P9a* does not show the massive over-expression seen in southern Africa [6]. The different patterns of gene duplication within the CYP6 cluster seen in different populations also suggest different haplotypes under selection. Further work to elucidate exactly how these duplications affect gene expression levels and resistance profiles may provide further information on this.

That other resistance associated CYP6 genes in the same gene cluster are differentially expressed among populations [6] also suggests that different haplotypes, underlying both allelic differences and gene expression profiles, may be under selection in different populations. This is consistent with the observation of different insecticide resistance profiles in different populations. For example Malawian *An*. *funestus* populations are resistant to pyrethroids and carbamates [32, 33] whereas Ugandan populations are resistant to pyrethroids and DDT but fully susceptible to carbamates [13, 34].

Overall, our results underlined the key role played by the CYP6 gene cluster on chromosome arm 2R, previously identified as the *rp1* pyrethroid resistance locus [25]. We found that multiple, independent selective sweeps have occurred spanning this CYP6 cluster in different parts of Africa.

Typically, because mosquito populations are resistant to multiple different insecticides it is difficult to establish which selective sweep is driven by a specific resistance mechanism and only further functional characterisation of the genes involved will help decipher this complexity. Similar findings, of multiple, independently selected haplotypes, have been reported for *An*. *gambiae* and *An*. *coluzzii* [4]. This strong variation highlights the fact that different resistance mechanisms are driving resistance to insecticides across the continent and thus design of resistance management strategies will need to be tailored to respective regions or countries.

Analysis of temporal changes in genomic diversity is a powerful method to detect selection. Previously, we used this method to show that scaled-up use of insecticide treated bed nets since 2002 in Malawi appears to have driven the selective sweep spanning CYP6 gene cluster on chromosome arm 2R [5]. Here, we expanded that analysis to show a similar recent selective sweep at the same locus in Mozambique. Similar insecticide-driven selection has been reported in *Drosophila* at the *CYP6G1* locus driving metabolic resistance to DDT [35] and in anopheline mosquitoes at insecticide target loci: the voltage gated sodium channel gene (target of pyrethroids and DDT) and a GABA-gated chloride channel subunit gene (target of dieldrin) [4].

Other populations such as Benin exhibited multiple selective sweeps. Indeed, our data showed major selective sweeps in Kpome, Benin around both the CYP6 cluster and the GSTe cluster. This population has been shown to be highly resistant to both class I and II pyrethroids, DDT and the carbamate bendiocarb [9]. That study also employed insecticide bioassays incorporating piperonyl butoxide (PBO; a synergist that inhibits cytochrome P450 activity) to infer that pyrethroid resistance was largely mediated by cytochrome P450s, while DDT resistance was not (*GSTe2* was suggested to mediate DDT resistance). The population genomic results are consistent with pyrethroids and DDT driving selective sweeps at both the CYP6 cluster the GST epsilon cluster. This is supported by the presence of a nearly fixed haplotype of the P450 *CYP6P9b* after gene conversion in Kpome whereas the 119F-GSTe2 haplotype associated with DDT resistance is fixed in this location [9]. Djouaka *et al*. also demonstrated carbamate resistance in the population. Carbamate resistance mechanisms are less well known in mosquitoes, including *An*. *funestus*. *CYP6AA1* has been shown to metabolise the pyrethroids permethrin and deltamethrin, as well as the carbamate bendiocarb [36]. Interestingly, we found that *CYP6AA1* appears to exist in multiple copies (along with partial copies of *CYP6AA2*) in the selected CYP6 cluster haplotype in Kpome, Benin. Whether this is associated with pyrethroid and/or carbamate resistance is not known and would benefit from further study. However, increased copy number does not appear to be associated with increased levels of gene expression in Benin [36]. The consistent association observed between reduced genetic diversity and over-expression of key detoxification genes such as *CYP6P9a/b* [6, 29], *GSTe2* [21] and *CYP9K1* (shown in [6] and in this study) suggests that metabolic resistance in malaria vectors is mainly driven by *cis*-regulatory changes, as recently demonstrated for *CYP6P9a/b* [6]. This also shows that assessing the *cis*-regulatory elements involved should facilitate the detection of resistance markers to design DNA-based assays to detect and track the spread of resistance in the field, as recently done for *CYP6P9a/b*.

## Conclusions

Analysis of genome-wide polymorphism in the major malaria vector *Anopheles funestus* elucidates the population history and structure of the species and identifies signatures of recent positive selection driven by insecticide use. The identification of multiple, independent selective sweeps at the same locus highlights the evolutionary plasticity of the species and its ability to evolve in response to vector control efforts. This strengthens the case for active resistance management strategies and the development of novel insecticides and alternative control strategies.

## Materials and Methods

### Collection of mosquitoes used in the study

The *An*. *funestus* FUMOZ laboratory colony is a multi-insecticide resistant colony derived from southern Mozambique [37], from which the reference genome assembly is derived. FANG is a fully insecticide susceptible colony derived from Angola [37].

Wild mosquitoes were sampled from 8 locations across the continental range of *An*. *funestus* between 2014 and 2016. Mosquitoes were sampled from the following countries: Benin (Kpome, 2015)[9]; Ghana (Obuasi, 2014)[38]; Cameroon (Mibellon, 2015) [39]; Uganda (Tororo, 2014) [34]; Democratic Republic of Congo [Kinshasa (2015), Mikalayi (2015] [12]; Malawi (Chikwawa, 2014) [33]; Mozambique (Palmeira, 2016) [40] and Zambia (Kaoma, 2013) further details are presented in S5 Table. In all cases, after obtaining the consent of village chiefs and house owners, blood fed adult female *An*. *funestus* mosquitoes resting indoors were collected from the ceilings and walls of houses using torches and aspirators between 06:00 a.m. and 12:00 p.m. Dead adult mosquitoes were transported (under DEFRA license PATH/125/2012) to the Liverpool School of Tropical Medicine for analysis. Samples collected in 2002 in Chikwawa, Malawi [5] and Morrumbene, Mozambique [5] were used to analyse temporal changes in genetic diversity.

### DNA extraction, sequence library preparation and sequencing

Genomic DNA (gDNA) was extracted from individual adult female mosquitoes using either the Qiagen DNeasy Blood and Tissue kit (Qiagen, Hilden, Germany) or the method of Livak [41]. It was quantified using Picogreen assays (Thermo-Fisher).

For pooled template whole genome sequencing (PoolSeq), equal quantities of gDNA were pooled from 40 individuals in all cases except Mikalayi, DRC, where due to fewer available samples, 29 individuals were pooled (S1 Table). Each pool was used to generate an Illumina TruSeq Nano DNA fragment library (insert size 350 bp). These (4 libraries per lane) were sequenced on an Illumina HiSeq 2500, using v4 chemistry to produce 2x125 bp paired-end reads. Library preparation and sequencing were carried out at the Centre for Genomic Research (CGR), University of Liverpool, UK.

Double-digest restriction site-associated DNA sequencing (ddRADseq) was carried out following the protocol of the White lab (protocol available to download from http://mosquitogenomics.org/protocols/). A minimum of 50 ng of genomic DNA for each sample was used to prepare double-digest Restriction-site Associated DNA (ddRAD) libraries, following a protocol modified from Peterson *et al*. [42]. The restriction enzymes *MluC1* and *NlaIII* (NEB, Ipswich, MA, USA) were used to digest DNA of individual mosquitoes, yielding RAD-tags of different sizes, to which short barcoded DNA adapters were ligated to enable the identification of reads belonging to each specimen. These fragments were purified, pooled and size-selected to select fragments of around 400 bp that were then amplified by PCR. The distribution of fragment sizes was checked on a BioAnalyzer (Agilent, Santa Clara, CA, USA) before sequencing. The library was sequenced on two lanes of an Illumina HiSeq 2000 (Illumina, San Diego, CA, USA). Sequencing was single-ended and read length was 101 bp (including an in-line barcode and restriction site). Sequencing was carried out at the Genomics Core Facility, University of California, Riverside, California.

### Analysis of pooled template DNA sequencing (PoolSeq) data

PoolSeq sequence reads were trimmed to remove sequenced Illumina adapters (matching >3 bp at the 3’ end) using cutadapt v1.2.1 [43] and low quality sequence (with a window quality score <20), using Sickle v1.200 [44]. After trimming, reads shorter than 10 bp were removed. If both reads of a pair passed this filter, each was included in either the R1 (forward reads) or R2 (reverse reads) file. If only one read of a pair passed this filter it was included in the R0 (unpaired reads) file. R1/R2 read pairs and R0 singleton reads were aligned to the reference genome sequence using bowtie2 v2.2.4 [45], with ‘sensitive-local’ alignment parameters and expected read pair orientation ‘fr’ and fragment size less than 500bp. Alignments were filtered to remove reads with mapping quality less than 10 using samtools [46] and to remove duplicate reads using picard tools’ ‘MarkDuplicates’ (http://broadinstitute.github.io/picard/). The distribution of coverage depth of all covered sites was calculated using samtools mpileup and custom scripts. These mpileup files were also used to identify regions of greater than expected coverage depth in order to identify copy number polymorphisms.

Estimation of population genetic indices was carried out using popoolation v1.2.2 and popoolation2 [47]. For estimation of intra-population indices, Samtools mpileup was used to generate files that were subsampled (with replacement) to a uniform coverage depth of 20x (10x for MWI 2014, where coverage depth was lower) using the popoolation script ‘subsample-pileup.pl’; sites with coverage depth less than 20 or greater than the 95^th^ centile of coverage depth were removed. These files were used to estimate pi, theta and Tajima’s D for windows of 50 kb moving in steps of 25 kb. To identify putative selective sweeps, the distribution of Tajima’s D values across all windows was calculated for each sample. Initial inspection of the data showed highly variable values on the X chromosome in some samples, so only the four autosomal chromosome arms were included in the genomic scan. Windows with Tajima’s D values in the lowest 0.1% were taken as the most likely to be under positive directional selection.

For estimation of pairwise inter-population divergence, Samtools mpileup was used to generate files from two different sample alignments, that were synchronised using the popoolation2 script ‘mpileup2sync.jar’ and subsampled to 20x coverage using ‘subsample-synchronized.pl’. Pairwise *F*_*ST*_ was calculated for each site and for windows of 50 kb moving in steps of 25 kb using ‘fst-sliding.pl’. Allele frequency differences were calculated using ‘snp-frequency-diff.pl’ and the significance of these differences calculated using ‘fisher-test.pl’. The highest 0.1% of pairwise *F*_*ST*_ values was used to identify the most divergent regions of the genome among populations.

For additional analyses of allele frequencies, variant calling was carried out on the mapping quality- and duplicate-filtered alignments using SNVer version 0.5.3, with default parameters [48]. SNPs were filtered to remove those with total coverage depth less than 10 or more the 95^th^ percentile for each sample as the allele frequency estimates could be inaccurate due to low coverage or misaligned paralogous sequence, respectively.

To define the historical relationships among populations using the PoolSeq whole genome data, reads for each population were aligned using BWA (v0.7.17-r1188) [49] to the *An*. *funestus* Afun1 genome assembly and sorted and deduplicated with Picard (v2.18.15, http://broadinstitute.github.io/picard). A multiple pileup file was created with samtools (v1.9) [46] mpileup from per-population bam files and used as input to VarScan (v2.4.3) [50]. Variants were called with a p-value threshold of 0.05 and normalised in bcftools [51]. Multiallelic sites were split into component alleles and removed if occurring within 20 bp of an indel. Finally, only SNPs were then retained for further analysis. The filtered VCF file was first converted into genobaypass format (read counts corresponding to major and minor reads per allele) using the R package poolfstat [52] and then into TreeMix format by custom scripts. TreeMix (v1.13) [53] was used to infer trees using allele counts from the pooled data without migration with SNPs combined into block of 1000 (-k 1000) for ten iterations, and the same tree topology was recovered each time. The topology for a representative run is presented, the drift parameter reflects the amount of genetic drift that has occurred between populations.

For detailed analysis of the selective sweep and complex molecular evolution at the CYP6 gene cluster on chromosome arm 2R, PoolSeq libraries were aligned to the 120 kb rp1 BAC sequence (accession PRJEB37305) containing this gene cluster. The reason for doing this was that the scaffold in genome assembly AfunF1 that contains the CYP6 gene cluster (KB119169) contains gaps within the cluster, making detailed analysis difficult. Alignments were visually inspected and inference of duplication was based on three linked lines of evidence: (i) increased coverage depth over part of the gene cluster, indicating more than one copy of the genome region; (ii) the presence of read pairs with anomalous relative orientations and insert lengths, indicating deletion, inversion or head-to-tail tandem orientation of duplicated genome regions; (iii) the presence of multiple apparently chimeric reads that span putative breakpoints and are clipped thereafter, or not, in the alignment. (ii) and (iii) are illustrated in S7 Fig. Exact breakpoints and the tandem organization of duplicated regions were confirmed by identifying matches between clipped sequences from one breakpoint and genomic sequences from the other, indicating that the clipped read is chimeric with part aligning adjacent to one breakpoint and part adjacent to the other and defining the breakpoints exactly (S7 Fig).

### Analysis of double-digest restriction site-associated DNA sequencing (ddRADseq) data

265,186,694 single-end reads were produced from two Illumina HiSeq lanes (134,082,619 reads for lane 1 and 131,104,075 reads for lane 2). After adding index sequences to the read headers, reads were de-multiplexed using the ‘process_radtags’ script from stacks v1.34 [54]. The de-multiplexed read sets were aligned to the reference genome assembly using bowtie2, v2.2.4 [45], with ‘sensitive’ alignment parameters. Samples with fewer than 250,000 reads aligned to the reference were removed from the analysis, leaving 39, 46, 44, 28 and 24 samples from Ghana, Cameroon, Uganda, Malawi and Zambia, respectively, for analysis.

Filtered aligned data were analysed using Stacks, version 1.34 [54]. ‘Stacks’ of sequence read coverage were defined for each sample using pstacks. A minimum of 3x coverage was required to define a stack. Then, all samples from each population were compared to define a non-redundant catalogue of stacks using cstacks. Stacks were matched based on genomic location. Finally, all samples from each population were compared back to this catalogue using Stacks. Again, stacks were matched based on genomic location.

Pairwise population divergence (*F*_*ST*_) was estimated using the stacks populations programme, with a minimum of 3x coverage per locus, minimum MAF of 1% and genotyped in at least 75% of individuals in each population. Pairwise AMOVA-*F*_*ST*_ was calculated, with a 50 kb window used for kernel-smoothing of *F*_*ST*_ estimates (for plotting).

Bayesian analysis of population structure was carried out using STRUCTURE version 2.3.4 [55]. The stacks populations programme was used to output a set of SNPs (one per RAD locus) with a minimum of 5x coverage, minimum MAF of 1% and genotyped in 100% of individuals in every population. An individual-based admixture model was applied to estimate the ancestry of each individual (n = 181) genotyped at 1280 SNP loci. A burn-in period of 10,000 generations and 20,000 Markov Chain Monte Carlo iterations were used, and 20 independent runs for each value of K (the number of ancestral clusters) from 2 to 10. Structure Harvester [56] was used to infer the most likely number of ancestral clusters (K) using Evanno’s method [57]. CLUMPP [58] was used to collate the data from all 20 replicate runs for each given K value, for plotting in R.

The same set of 1280 SNPs in 181 individuals was used to estimate overall *F*_*ST*_ using GENEPOP [59] and to generate a PCA plot using the R package ADEGENET [60].

### Reference genome assembly scaffolds

The reference sequence used comprised 1392 *Anopheles funestus* assembled scaffolds (assembly AfunF1; GenBank assembly identifier GCA_000349085.1; GenBank WGS project identifier APCI01) downloaded from VectorBase (www.vectorbase.org) and a sequence representing the *An*. *funestus* mitochondrial genome (GenBank accession number DQ146364.1). In order to display population genetic indices in a whole-genome context, the 1392 scaffolds of AfunF1 were ordered relative to *Anopheles gambiae* (assembly AgamP4) chromosomes, in order to modify the coordinates of SNPs/windows etc. to display them on a single plot. Nucmer, from MUMmer v3.0 [61] was used to align *An*. *funestus* (assembly AfunF1) scaffolds to *Anopheles gambiae* chromosomes (unplaced *An*. *gambiae* scaffolds were excluded). Nucmer alignment placed 644 of the 1392 scaffolds (46%). The total length of these was 217,255,185 bp, of a total of 225,223,604 bp (96%), the vast majority of the total genome sequence. The final file contained all 1392 scaffolds ordered according to Agam (with 2L and 3R transposed), including the unplaced scaffolds at the end.

### Analysis of gene conversion between *CYP6P9a* and *CYP6P9b*

To identify evidence of gene conversion between the paralogous *CYP6P9a* and *b* genes, we searched the GenBank nucleotide sequence database using the text terms “*CYP6P9a*” and “*CYP6P9b*”. Spurious matches were removed to leave 280 *CYP6P9a* and 118 *CYP6P9b* sequences for analysis. A full-length FUMOZ coding sequence for each gene from VectorBase brought the total number of sequences to 400. Details of the sequences are shown in S4 Table. The 400 sequences were aligned using MUSCLE [62], implemented within the sequence and alignment editor Seaview [63]. 348/400 sequences had sequence upstream of the translation start codon. This was trimmed from all sequences. 162/400 sequences did not include the 3’ end of the gene but stopped at nucleotide position 1464 of both *CYP6P9a* (AFUN015792) and *CYP6P9b* (AFUN015889). All sequences were trimmed to this position. 350/400 sequences included the intron. This was trimmed from all sequences. The trimmed alignment was 1464 nucleotides long.

This alignment (excluding sites with alignment gaps) was used to generate a multidimensional scaling (MDS) plot and a Neighbour-Joining phylogeny from pairwise divergence among all 400 sequences (281 *CYP6P9a*; 119 *CYP6P9b*). The MDS plot was generated in R. The Neighbour-Joining phylogeny was generated using PHYLIP [64], implemented within Seaview [63], with a Kimura 2-parameter distance correction and 1000 bootstrap replicates.

### Availability of data and materials

All genomic datasets are available from the European Nucleotide Archive. Pooled template whole genome sequencing data are available under study accessions PRJEB13485 (Malawi 2002 and Malawi 2014), PRJEB24384 (Ghana, Benin, Cameroon and Uganda) and PRJEB35040 (Mozambique 2002, 2016; DRC-Kinshasa and Mikalayi). The rp1 BAC sequence is available under study accession PRJEB37305.

## Supporting information

S1 Fig Genome-wide distribution of Tajima’s D in 8 African populations of *Anopheles funestus*. Each point is Tajima’s D calculated for a 50 kb window (moving in increments of 25 kb) using PoolSeq alignments. The highest and lowest 1% and 0.1% of Tajima’s D values are shown in orange (1%) and red (0.1%). The populations are (A) Obuasi, Ghana (GHA); (B) Kpome, Benin (BEN); (C) Mibellon, Cameroon (CMR); (D) Tororo, Uganda (UGA); (E) Kinshasa, Democratic Republic of Congo (COD-K); (F) Mikalayi, DRC (COD-M); (G) Chikwawa, Malawi (MWI); (H) Palmeira, Mozambique (MOZ). The selective sweep near 31 Mbp (on 2R) is the CYP6 cluster in scaffold KB669169. That near 81 Mbp (on 2L) in Benin (panel B) is the GST epsilon cluster on scaffold KB669036. That near 88 Mbp (on 2L) in all populations is on scaffold KB668697 and that near 111 Mbp (on 3R) is on scaffold KB668905.
(TIFF)

S2 Fig Genome-wide distribution of pairwise genetic differentiation (*F*_*ST*_) between African populations of *Anopheles funestus*. Each point is *F*_*ST*_ calculated for a 50 kb window (moving in increments of 25 kb) using ddRADseq alignments. The highest 1% and 0.1% of *F*_*ST*_ values are shown in orange (1%) and red (0.1%). The populations are: Obuasi, Ghana (GHA); Mibellon, Cameroon (CMR); Tororo, Uganda (UGA); Chikwawa, Malawi (MWI); Kaoma, Zambia (ZMB). Compared populations are indicated on each panel with (A) showing GHA_vs_CMR, (B) is GHA_vs_UGA, (C) is GHA_vs_MWI, (D) is GHA_vs_ZMB, (E) is CMR_vs_UGA, (F) is CMR_vs_MWI, (G) is CMR_vs_ZMB, (H) is UGA_vs_MWI, (I) is UGA_vs_ZMB, (J) is MWI_vs_ZMB. The extreme genetic differentiation observed near 31 Mbp (on 2R) coincides with the CYP6 cluster on scaffold KB669169. Several regions of elevated *F*_*ST*_ between Ghana and other populations can be seen on 2R.
(TIFF)

S3 Fig Complex molecular evolution in the CYP6 gene cluster on chromosome arm 2R. Sequence alignment views from the Integrative Genomics Viewer (IGV) showing evidence of duplications in the CYP6 gene cluster. Alignments are of PoolSeq data to the rp1 BAC (accession PRJEB37305). In each panel, the top track shows coverage depth (coloured bars indicate sites with 100% non-FUMOZ nucleotides, indicating non-FUMOZ haplotypes). The middle track shows read pair alignments (rectangles are reads, joined as read pairs by thin lines; non-grey pairs are in unexpected relative orientation/distance to one another). The lower track shows the genes (blue), from left to right: *CYP6AA1*, *CYP6AA2*, two carboxylesterases, *CYP6P15P*, *CYP6P9a*, *CYP6P9b*, *CYP6P5*, *CYP6P4a*, *CYP6P4b*, *CYP6P1*, *CYP6P2*, *CYP6AD1*. Red boxes enclose identified duplications and the blue box encloses the deletion. (A) A large duplication spanning the entire gene cluster (rp1:10311–71182) and one spanning *CYP6AA1* and part of *CYP6AA2* (rp1:17910–24836) in Ghana. (B) A duplication spanning *CYP6P5* and *CYP6P4a* (rp1:46407–52668) in Ghana. (C) A duplication spanning *CYP6AA1* and part of *CYP6AA2* (rp1:18643–25686) in Benin. (D) Duplications spanning *CYP6AA1* and part of *CYP6AA2* (rp1:19133–25617) and one spanning *CYP6AA1*, *CYP6AA2*, 2x carboxylesterases, *CYP6P15P* and partial *CYP6P9a* (rp1:19043–35875) in Cameroon. (E) A duplication spanning *CYP6P5* (rp1:46771–50640) and containing a deletion (rp1:46864–49106) in Kinshasa, Democratic Republic of Congo.
(TIFF)

S4 Fig Analysis of the selected haplotype at the GST epsilon gene cluster on chromosome arm 2R in Benin. (A) IGV alignment view showing the entire gene cluster. Coverage depth is indicated at the top and aligned reads below. Genes are shown at the bottom (blue) and their orientations indicated with arrows. Gene names are abbreviated (e.g. ‘e3’ is GSTe3). Genomic location on scaffold KB669036 (approximately between 98,000 and 108,000) is indicated at the top. The three vertical arrows at the top mark the positions of (from left to right) the transposon insertion shown in (B), the 1 bp deletion shown in (C) and the putative duplication in *GSTe6* shown in (E). (B) A transposon insertion upstream of *GSTe2* (position indicated by a vertical dotted line). The target site (TTAA) is indicated. Non-grey reads indicate members of a read pair for which their mate aligns elsewhere in the genome (this pattern, with all of these reads facing the putative insertion site is characteristic of a transposon insertion). In the blue (partial) gene models at the bottom, thinner parts indicate un-translated regions and thicker parts coding regions. (C) Alignment showing the 1 bp deletion (G) in Benin sequence reads relative to the FUMOZ reference (shown at the bottom). The transcription start site (TSS) of *GSTe2* is indicated. (D) Relative frequencies of T (red) and C (blue) bases in codon 119 of *GSTe2*. *GSTe2*-L119F is a DDT resistance marker. Codon CTT (Leucine) is the susceptible allele and TTT (Phenylalanine) is the resistant allele, present in 98% of aligned reads in Benin, 31% in Ghana and 27% in Cameroon. The preceding codon (ATT, Isoleucine) is also shown. (E) Evidence of duplication within *GSTe6* in the Benin selected haplotype. Coverage depth is indicated at the top and aligned reads below (here shown with thin lines linking each read pair). Green reads are discordant pairs, in an orientation suggesting tandem duplication. Multi-coloured parts of reads are sections trimmed during alignment, and demarcate breakpoints (indicated by vertical dotted lines).
(TIFF)

S5 Fig Reduced genetic diversity and over-expression of *CYP9K1* in Uganda. Kernel-smoothed minor allele frequency (MAF, coloured line, left axis) and gene expression, as fragments per kilobase per million mapped reads (FPKM, black bar, right axis, from [6]) across the *CYP9K1* (AFUN007549) and neighbouring genes on the X chromosome. The position (pos., in kb) is on scaffold KB668367. Patterns seen in (A) Uganda; (B) Ghana; (C) Cameroon and (D) Malawi suggest a selective sweep associated with over-expression of *CYP9K1* in Uganda.
(TIFF)

S6 Fig Detailed analysis of copy number variation spanning *CYP9K1* in Uganda. (A) Screenshot of the Integrative Genomics Viewer (IGV), showing the alignment of PoolSeq data from Uganda at and upstream of the *CYP9K1* gene (blue shape at the lower left). The putative transcription start site (TSS) at position KB668367:175642 is indicated. The track at the top of the panel summarises coverage depth, indicating the change from high (left) to low (right). The middle track shows individual sequence reads (grey rectangles) aligned to the reference sequence. Read pairs are linked by thin lines. Dark grey and cyan reads indicate discordant read pairs where the other read of the pair is aligned to a different scaffold in the genome assembly. This pattern, seen around position KB668367:176475, along with read clipping around this position and a small peak seen in the coverage depth track, are characteristic of the presence of a transposon insertion in the sequenced reads that is absent from the reference sequence. (B) Detailed analysis of clipped reads. IGV screenshot showing reads from discordant pairs (pink and green) and clipped reads (clipped regions are colour-coded by their bases–red, blue, green, orange) seen left of KB668367:176470 (‘clipped 1’) and right of KB668367:176479 (‘clipped 2’) and KB668367:176606 (‘clipped 3’). (C) Nucleotide sequences clipped (blue) from reads aligned to this putative breakpoint region. The GAAATTTG motif (in bold) was present in, but not clipped from, reads aligned in both directions and is a putative target site duplication causing a small peak in coverage depth, characteristic of a sequenced transposon. Underlined regions indicate putative terminal inverted repeats.
(TIFF)

S7 Fig Detection of structural variation using paired-end next generation sequencing reads. (A) Detection of a transposon insertion in the sequenced sample that is absent from the reference genome. The reference genome is shown in grey and sequencing reads shown as arrows. Between the two vertical dotted lines is a target sequence (in this case TTAA). Reads (in peach) align up to and including this sequence, then are clipped during alignment (clipped regions in pale blue) as clipped sequence represents transposon rather than flanking genomic sequence. The inserted transposon is shown below (in pale blue with target site duplications at each end). Clipped sequence can be used to search the genome and may match transposon(s) inserted elsewhere in the reference genome (shown on the right). Dark red and dark blue reads are from discordant read pairs in which one read aligns elsewhere in the genome (to a transposon elsewhere in the reference genome, shown on the right). (B) Detection of regions present in the reference genome that are deleted from the sequenced sample. Vertical dotted lines represent breakpoints (edges of the deletion). Reads (in peach) align up to these then are clipped during alignment (in white). Clipped sequence may align to the flanking sequence on the other side of the deleted region. Dark red reads indicate a discordant read pair (pair is joined by a thin black line), fully aligned in the correct orientation but with too large a gap between them. (C) Detection of tandem duplications in the sequenced sample that exist in only one copy in the reference genome. First is a reference containing a tandem duplication (each copy shown in pale green). Reads align across the breakpoint (where the two copies meet, head-to-tail) and pairs spanning the breakpoint are concordant. Second is a reference with only one copy of this duplication. If the sequenced sample contains a duplication, chimeric reads (derived from the head-to-tail breakpoint) will exist that align up to the edge of the duplicated region (aligned parts in peach, breakpoints indicated by vertical dotted lines) and be clipped thereafter (in pale green, as these parts will match the other end of the duplicated region). Also, concordant read pairs spanning the head-to-tail breakpoint (in peach in the first diagram) will appear discordant when only one copy is represented in the reference genome (in dark red in the second diagram).
(TIFF)

S1 Table Details of PoolSeq libraries. (PDF)

S2 Table Summary statistics of PoolSeq sequence alignments. (PDF)

S3 Table Alignment coverage depth profiles (centiles of coverage depth, c), number of sites in the coverage depth range allowed for SNP-calling (>10x, <c95) and number of SNP variants identified for each PoolSeq samples. (PDF)

S4 Table *CYP6P9a* and *CYP6P9b* sequences included in an analysis of gene conversion. (PDF)

S5 Table Details of Mosquito collection sites across Africa. (PDF)
