# Supplementary figures and images for: An Africa-wide genomic evolution of insecticide resistance in the malaria vector Anopheles funestus involves selective sweeps, copy number variations, gene conversion and transposons
Source: PLoS Genet. 2020 Jun 4;16(6):e1008822. doi: 10.1371/journal.pgen.1008822 (PMC7297382; doi:10.1371/journal.pgen.1008822)

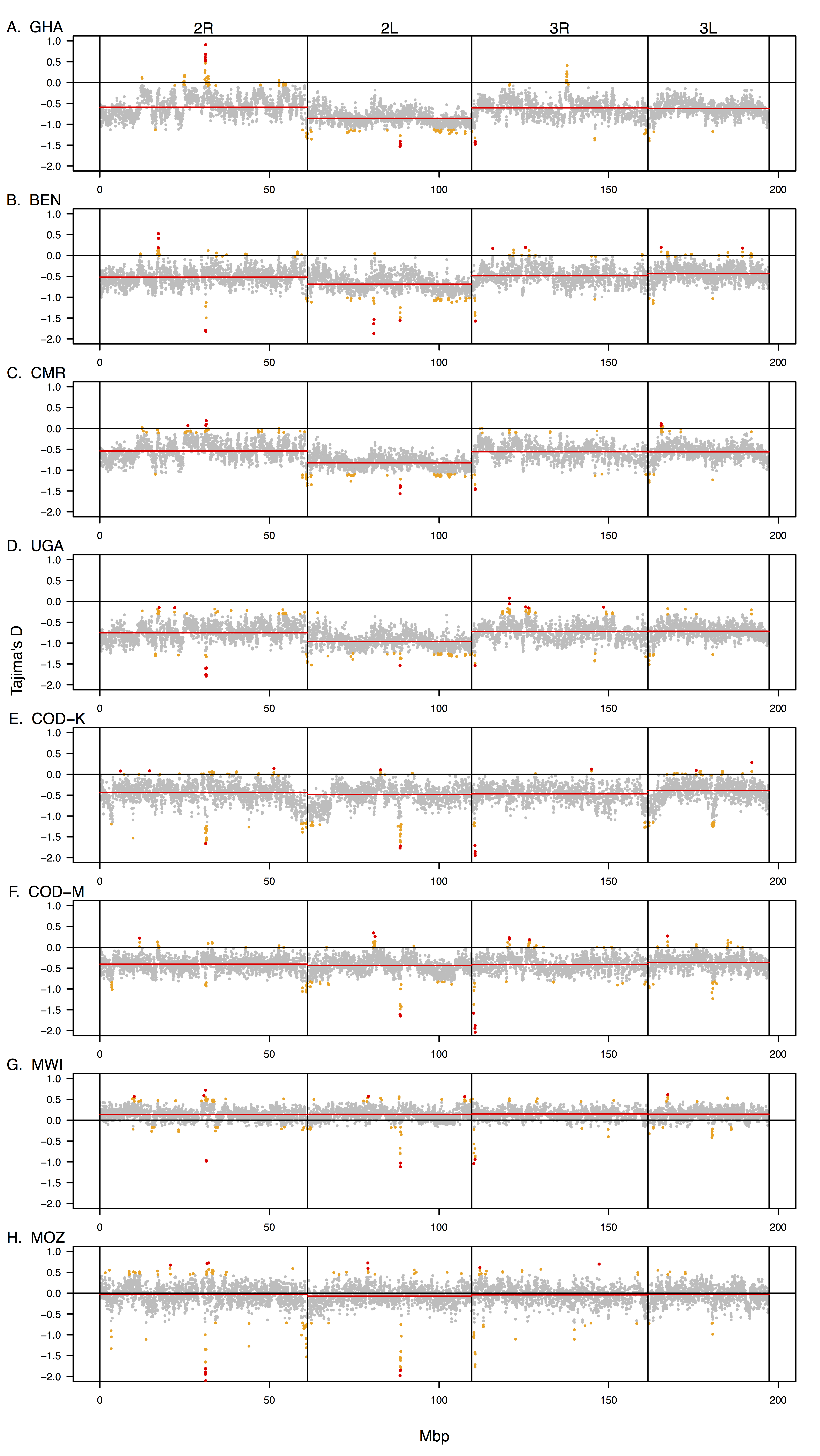

Supplement: S1 Fig — Each point is Tajima’s D calculated for a 50 kb window (moving in increments of 25 kb) using PoolSeq alignments. The highest and lowest 1% and 0.1% of Tajima’s D values are shown in orange (1%) and red (0.1%). The populations are (A) Obuasi, Ghana (GHA); (B) Kpome, Benin (BEN); (C) Mibellon, Cameroon (CMR); (D) Tororo, Uganda (UGA); (E) Kinshasa, Democratic Republic of Congo (COD-K); (F) Mikalayi, DRC (COD-M); (G) Chikwawa, Malawi (MWI); (H) Palmeira, Mozambique (MOZ). The selective sweep near 31 Mbp (on 2R) is the CYP6 cluster in scaffold KB669169. That near 81 Mbp (on 2L) in Benin (panel B) is the GST epsilon cluster on scaffold KB669036. That near 88 Mbp (on 2L) in all populations is on scaffold KB668697 and that near 111 Mbp (on 3R) is on scaffold KB668905. (TIFF) [file pgen.1008822.s001.tiff]

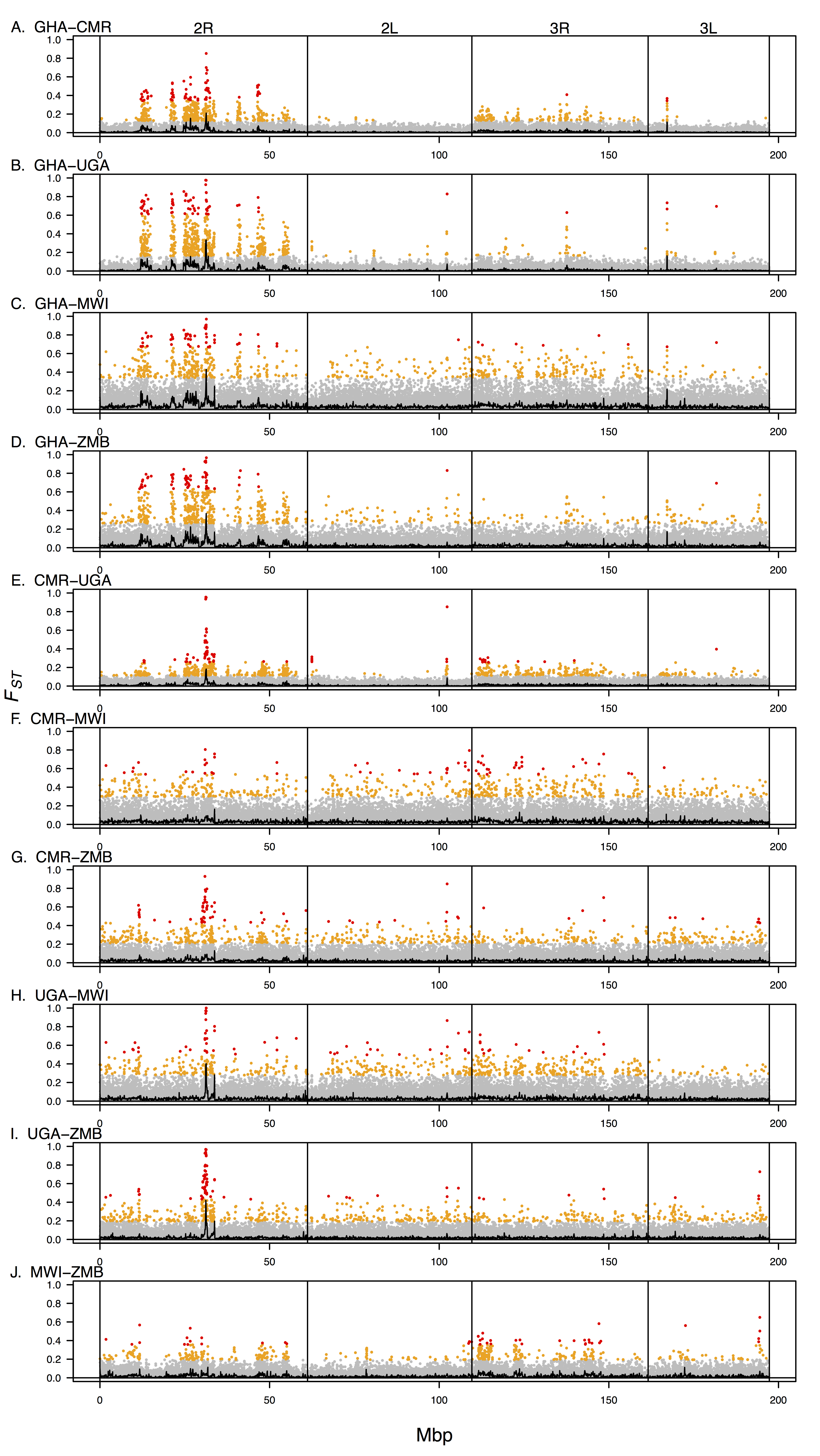

Supplement: S2 Fig — Each point is FST calculated for a 50 kb window (moving in increments of 25 kb) using ddRADseq alignments. The highest 1% and 0.1% of FST values are shown in orange (1%) and red (0.1%). The populations are: Obuasi, Ghana (GHA); Mibellon, Cameroon (CMR); Tororo, Uganda (UGA); Chikwawa, Malawi (MWI); Kaoma, Zambia (ZMB). Compared populations are indicated on each panel with (A) showing GHA_vs_CMR, (B) is GHA_vs_UGA, (C) is GHA_vs_MWI, (D) is GHA_vs_ZMB, (E) is CMR_vs_UGA, (F) is CMR_vs_MWI, (G) is CMR_vs_ZMB, (H) is UGA_vs_MWI, (I) is UGA_vs_ZMB, (J) is MWI_vs_ZMB. The extreme genetic differentiation observed near 31 Mbp (on 2R) coincides with the CYP6 cluster on scaffold KB669169. Several regions of elevated FST between Ghana and other populations can be seen on 2R. (TIFF) [file pgen.1008822.s002.tiff]

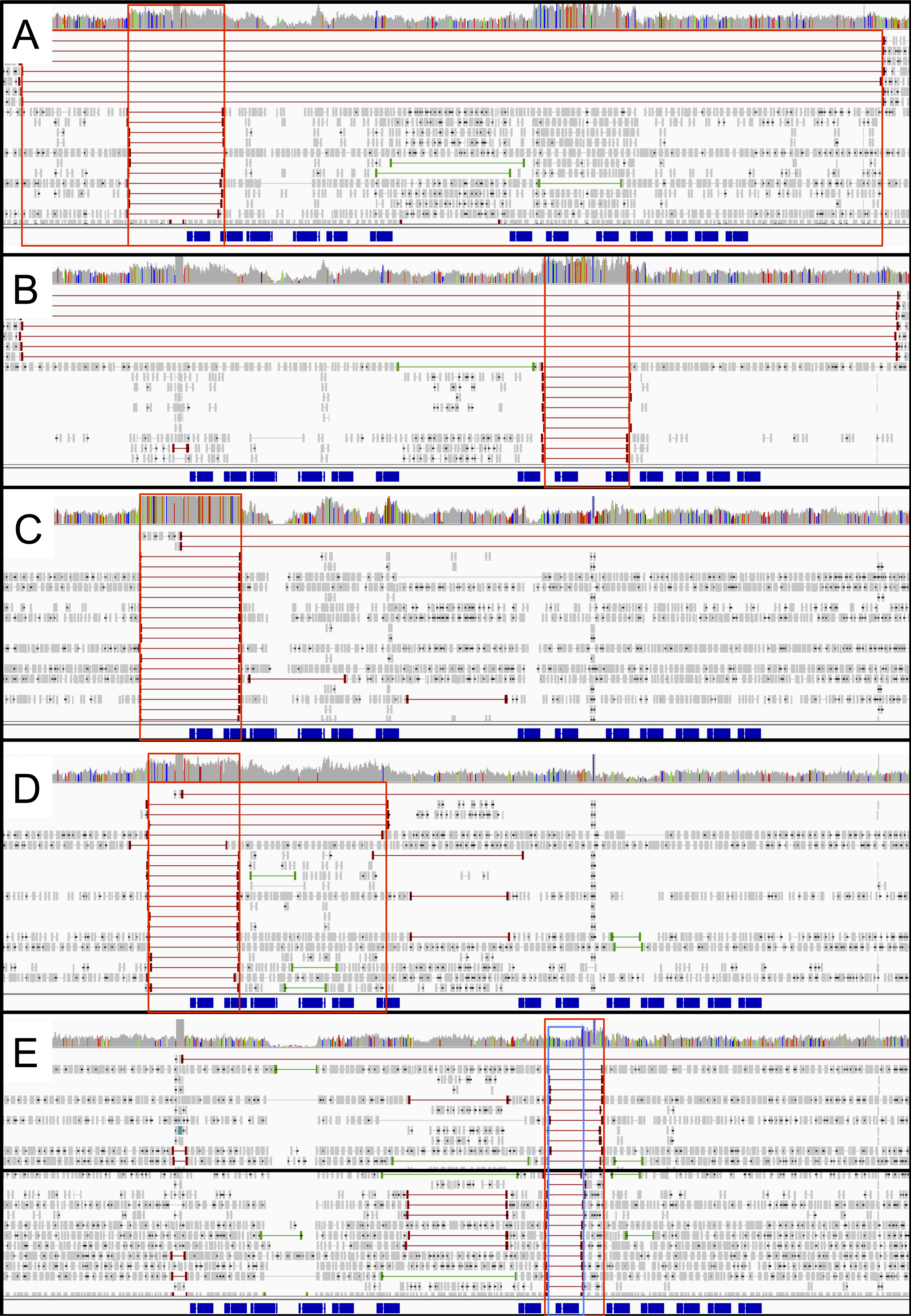

Supplement: S3 Fig — Sequence alignment views from the Integrative Genomics Viewer (IGV) showing evidence of duplications in the CYP6 gene cluster. Alignments are of PoolSeq data to the rp1 BAC (accession PRJEB37305). In each panel, the top track shows coverage depth (coloured bars indicate sites with 100% non-FUMOZ nucleotides, indicating non-FUMOZ haplotypes). The middle track shows read pair alignments (rectangles are reads, joined as read pairs by thin lines; non-grey pairs are in unexpected relative orientation/distance to one another). The lower track shows the genes (blue), from left to right: CYP6AA1, CYP6AA2, two carboxylesterases, CYP6P15P, CYP6P9a, CYP6P9b, CYP6P5, CYP6P4a, CYP6P4b, CYP6P1, CYP6P2, CYP6AD1. Red boxes enclose identified duplications and the blue box encloses the deletion. (A) A large duplication spanning the entire gene cluster (rp1:10311–71182) and one spanning CYP6AA1 and part of CYP6AA2 (rp1:17910–24836) in Ghana. (B) A duplication spanning CYP6P5 and CYP6P4a (rp1:46407–52668) in Ghana. (C) A duplication spanning CYP6AA1 and part of CYP6AA2 (rp1:18643–25686) in Benin. (D) Duplications spanning CYP6AA1 and part of CYP6AA2 (rp1:19133–25617) and one spanning CYP6AA1, CYP6AA2, 2x carboxylesterases, CYP6P15P and partial CYP6P9a (rp1:19043–35875) in Cameroon. (E) A duplication spanning CYP6P5 (rp1:46771–50640) and containing a deletion (rp1:46864–49106) in Kinshasa, Democratic Republic of Congo. (TIFF) [file pgen.1008822.s003.tiff]

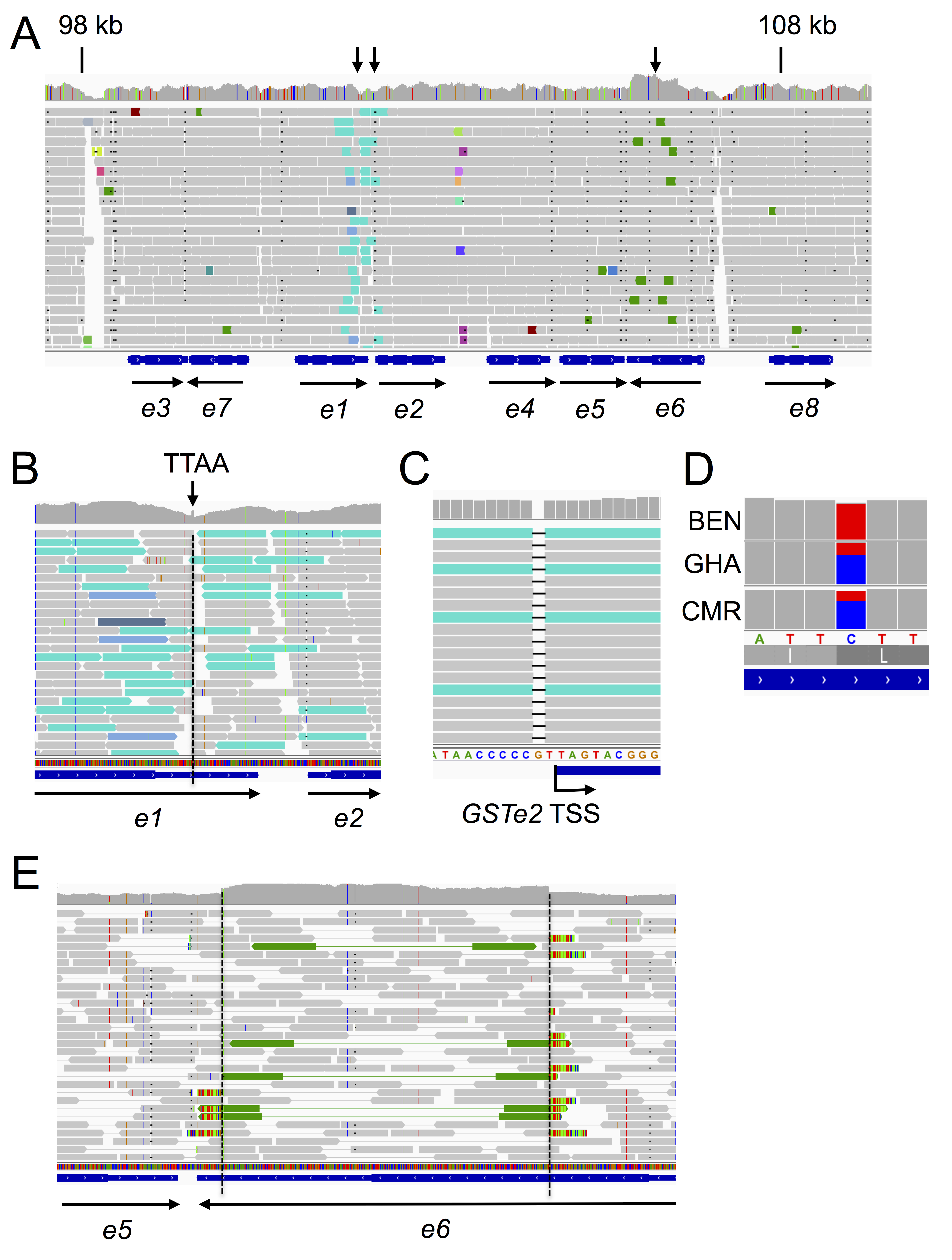

Supplement: S4 Fig — (A) IGV alignment view showing the entire gene cluster. Coverage depth is indicated at the top and aligned reads below. Genes are shown at the bottom (blue) and their orientations indicated with arrows. Gene names are abbreviated (e.g. ‘e3’ is GSTe3). Genomic location on scaffold KB669036 (approximately between 98,000 and 108,000) is indicated at the top. The three vertical arrows at the top mark the positions of (from left to right) the transposon insertion shown in (B), the 1 bp deletion shown in (C) and the putative duplication in GSTe6 shown in (E). (B) A transposon insertion upstream of GSTe2 (position indicated by a vertical dotted line). The target site (TTAA) is indicated. Non-grey reads indicate members of a read pair for which their mate aligns elsewhere in the genome (this pattern, with all of these reads facing the putative insertion site is characteristic of a transposon insertion). In the blue (partial) gene models at the bottom, thinner parts indicate un-translated regions and thicker parts coding regions. (C) Alignment showing the 1 bp deletion (G) in Benin sequence reads relative to the FUMOZ reference (shown at the bottom). The transcription start site (TSS) of GSTe2 is indicated. (D) Relative frequencies of T (red) and C (blue) bases in codon 119 of GSTe2. GSTe2-L119F is a DDT resistance marker. Codon CTT (Leucine) is the susceptible allele and TTT (Phenylalanine) is the resistant allele, present in 98% of aligned reads in Benin, 31% in Ghana and 27% in Cameroon. The preceding codon (ATT, Isoleucine) is also shown. (E) Evidence of duplication within GSTe6 in the Benin selected haplotype. Coverage depth is indicated at the top and aligned reads below (here shown with thin lines linking each read pair). Green reads are discordant pairs, in an orientation suggesting tandem duplication. Multi-coloured parts of reads are sections trimmed during alignment, and demarcate breakpoints (indicated by vertical dotted lines). (TIFF) [file pgen.1008822.s004.tiff]

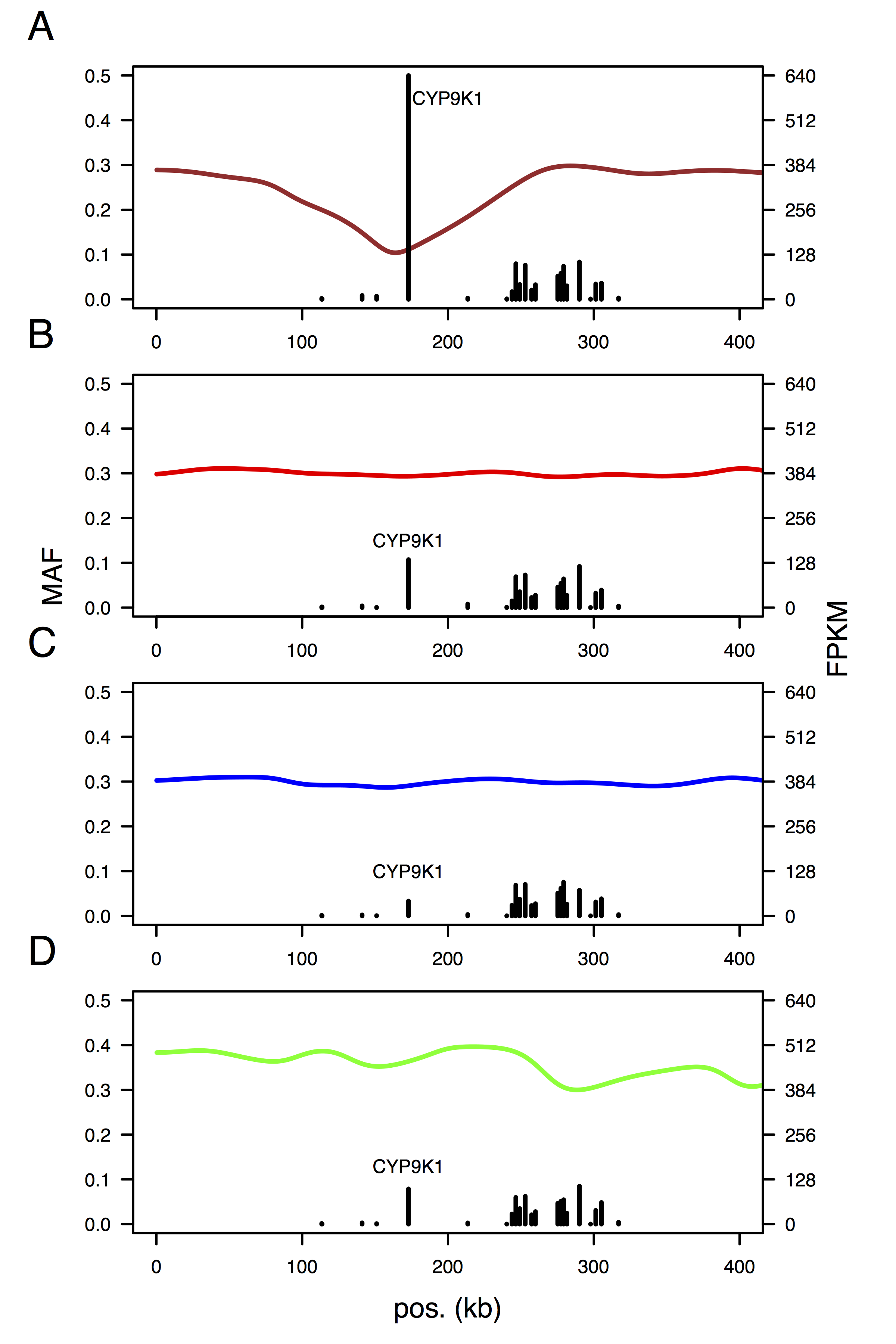

Supplement: S5 Fig — Kernel-smoothed minor allele frequency (MAF, coloured line, left axis) and gene expression, as fragments per kilobase per million mapped reads (FPKM, black bar, right axis, from [6]) across the CYP9K1 (AFUN007549) and neighbouring genes on the X chromosome. The position (pos., in kb) is on scaffold KB668367. Patterns seen in (A) Uganda; (B) Ghana; (C) Cameroon and (D) Malawi suggest a selective sweep associated with over-expression of CYP9K1 in Uganda. (TIFF) [file pgen.1008822.s005.tiff]

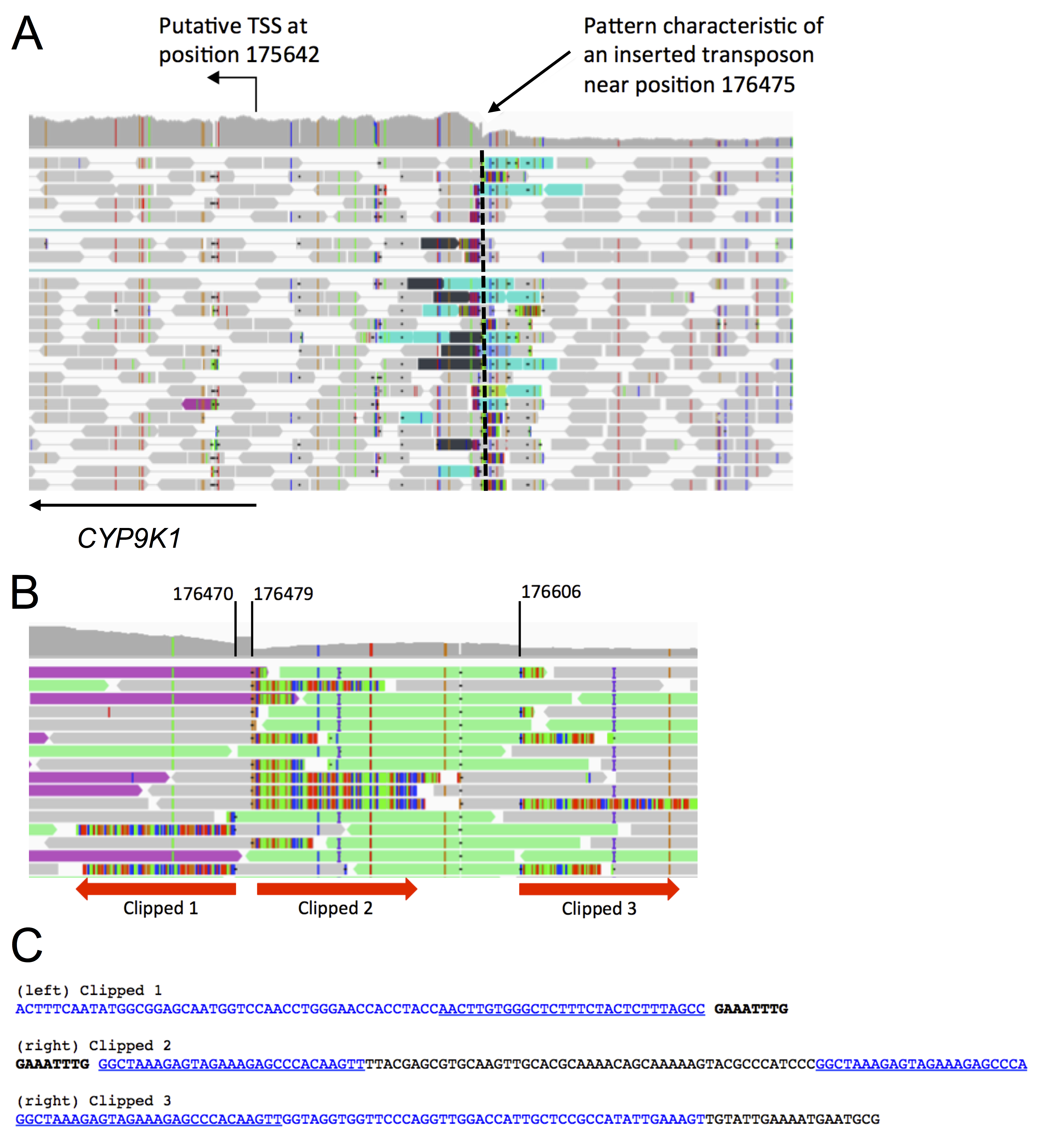

Supplement: S6 Fig — (A) Screenshot of the Integrative Genomics Viewer (IGV), showing the alignment of PoolSeq data from Uganda at and upstream of the CYP9K1 gene (blue shape at the lower left). The putative transcription start site (TSS) at position KB668367:175642 is indicated. The track at the top of the panel summarises coverage depth, indicating the change from high (left) to low (right). The middle track shows individual sequence reads (grey rectangles) aligned to the reference sequence. Read pairs are linked by thin lines. Dark grey and cyan reads indicate discordant read pairs where the other read of the pair is aligned to a different scaffold in the genome assembly. This pattern, seen around position KB668367:176475, along with read clipping around this position and a small peak seen in the coverage depth track, are characteristic of the presence of a transposon insertion in the sequenced reads that is absent from the reference sequence. (B) Detailed analysis of clipped reads. IGV screenshot showing reads from discordant pairs (pink and green) and clipped reads (clipped regions are colour-coded by their bases–red, blue, green, orange) seen left of KB668367:176470 (‘clipped 1’) and right of KB668367:176479 (‘clipped 2’) and KB668367:176606 (‘clipped 3’). (C) Nucleotide sequences clipped (blue) from reads aligned to this putative breakpoint region. The GAAATTTG motif (in bold) was present in, but not clipped from, reads aligned in both directions and is a putative target site duplication causing a small peak in coverage depth, characteristic of a sequenced transposon. Underlined regions indicate putative terminal inverted repeats. (TIFF) [file pgen.1008822.s006.tiff]

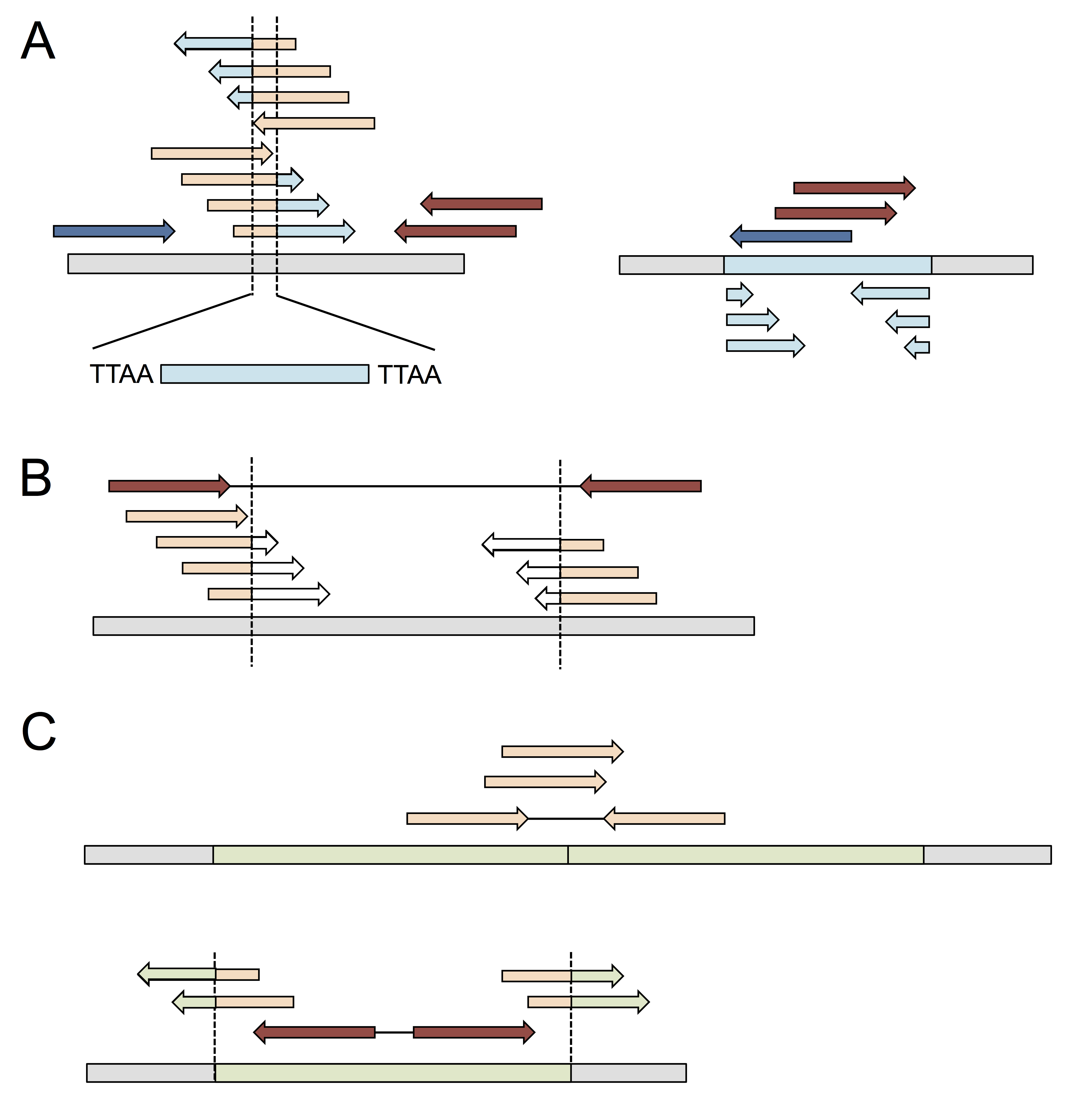

Supplement: S7 Fig — (A) Detection of a transposon insertion in the sequenced sample that is absent from the reference genome. The reference genome is shown in grey and sequencing reads shown as arrows. Between the two vertical dotted lines is a target sequence (in this case TTAA). Reads (in peach) align up to and including this sequence, then are clipped during alignment (clipped regions in pale blue) as clipped sequence represents transposon rather than flanking genomic sequence. The inserted transposon is shown below (in pale blue with target site duplications at each end). Clipped sequence can be used to search the genome and may match transposon(s) inserted elsewhere in the reference genome (shown on the right). Dark red and dark blue reads are from discordant read pairs in which one read aligns elsewhere in the genome (to a transposon elsewhere in the reference genome, shown on the right). (B) Detection of regions present in the reference genome that are deleted from the sequenced sample. Vertical dotted lines represent breakpoints (edges of the deletion). Reads (in peach) align up to these then are clipped during alignment (in white). Clipped sequence may align to the flanking sequence on the other side of the deleted region. Dark red reads indicate a discordant read pair (pair is joined by a thin black line), fully aligned in the correct orientation but with too large a gap between them. (C) Detection of tandem duplications in the sequenced sample that exist in only one copy in the reference genome. First is a reference containing a tandem duplication (each copy shown in pale green). Reads align across the breakpoint (where the two copies meet, head-to-tail) and pairs spanning the breakpoint are concordant. Second is a reference with only one copy of this duplication. If the sequenced sample contains a duplication, chimeric reads (derived from the head-to-tail breakpoint) will exist that align up to the edge of the duplicated region (aligned parts in peach, breakpoints indicated [file pgen.1008822.s007.tiff]
