# Supplementary material for: An Africa-wide genomic evolution of insecticide resistance in the malaria vector Anopheles funestus involves selective sweeps, copy number variations, gene conversion and transposons
Source: PLoS Genet. 2020 Jun 4;16(6):e1008822. doi: 10.1371/journal.pgen.1008822 (PMC7297382; doi:10.1371/journal.pgen.1008822)
Supplement: S1 Table — (PDF) [file pgen.1008822.s008.pdf]

Sample name	Pool size	Untrimmed reads	Trimmed reads	R1/R2 pairs [1]	R0 reads (%) 2
GHA-Obuasi-2014	40	101,074,972	100,593,664	50,069,251	455,162 (0.45%)
BEN-Kpome-2015	40	99,684,172	99,049,555	49,319,143	411,269 (0.42%)
CMR-Mebellom-2014	40	99,120,016	98,674,983	49,123,913	427,157 (0.43%)
COD-Kinshasa-2015	40	109,046,082	107,938,818	53,429,538	1,079,742 (1.00%)
COD-Mikalayi-2015	29	109,482,994	108,730,737	54,125,350	480,037 (0.44%)
UGA-Tororo-2014	40	97,365,064	96,991,083	48,315,549	359,985 (0.37%)
MWI-Chikwawa-2014	40	91,499,782	90,976,162	45,240,390	495,382 (0.54%)
MWI-Chikwawa-2002	40	100,946,514	100,344,549	49,951,213	442,123 (0.44%)
MOZ-Manhica-2016	40	169,986,238	168,020,763	83,053,393	1,913,977 (1.14%)
MOZ-Morrumbene-2002	40	104,122,630	103,013,413	51,293,356	426,701 (0.41%)

[1] Forward (R1) and reverse (R2) read pairs after trimming.
[2] Reads unpaired after trimming (% of total trimmed reads).
