# Supplementary material for: An Africa-wide genomic evolution of insecticide resistance in the malaria vector Anopheles funestus involves selective sweeps, copy number variations, gene conversion and transposons
Source: PLoS Genet. 2020 Jun 4;16(6):e1008822. doi: 10.1371/journal.pgen.1008822 (PMC7297382; doi:10.1371/journal.pgen.1008822)
Supplement: S2 Table — (PDF) [file pgen.1008822.s009.pdf]

| Sample ID | Reads to align (R1+R2+R0) | Aligned reads (%) [1] | Aligned reads, filtered (%) [1,2] | Aligned in pair (%) [3] | Properly paired (%) [3,4] | Singleton (%) [3] |
|---|---|---|---|---|---|---|
| GHA-Obuasi-2014 | 100,593,664 | 61,708,566 (61%) | 56,122,278 (56%) | 55,690,966 (99%) | 48,551,862 (87%) | 260,831 (0.5%) |
| BEN-Kpome-2015 | 99,049,555 | 77,760,764 (79%) | 70,065,274 (71%) | 69,590,103 (99%) | 65,309,148 (93%) | 250,733 (0.4%) |
| CMR-Mebellom-2014 | 98,674,983 | 60,566,286 (61%) | 55184243 (56%) | 54,726,342 (99%) | 47,294,648 (86%) | 280,747 (0.5%) |
| COD-Kinshasa-2015 | 107,938,818 | 63,691,312 (59%) | 56,850,352 (53%) | 55,619,705 (98%) | 49,126,540 (86%) | 803,615 (1.4%) |
| COD-Mikalayi-2015 | 108,730,737 | 88,874,076 (82%) | 80,470,521 (74%) | 79,871,664 (99%) | 71,656,064 (89%) | 320,287 (0.4%) |
| UGA-Tororo-2014 | 96,991,083 | 78,309,572 (81%) | 71,449,749 (74%) | 70,908,959 (99%) | 61,732,826 (86%) | 324,735 (0.5%) |
| MWI-Chikwawa-2014 | 90,976,162 | 30,929,682 (34%) | 28,399,605 (31%) | 28,158,651 (99%) | 24,343,506 (86%) | 155,992 (0.5%) |
| MWI-Chikwawa-2002 | 100,344,549 | 82,126,137 (82%) | 74,115,352 (74%) | 73,528,738 (99%) | 67,567,770 (91%) | 328,021 (0.4%) |
| MOZ-Manhica-2016 | 168,020,763 | 75,930,322 (45%) | 63,776,575 (38%) | 62,922,778 (99%) | 54,270,794 (85%) | 293,960 (0.5%) |
| MOZ-Morrumbene-2002 | 103,013,413 | 74,942,373 (73%) | 67,568,570 (66%) | 66,783,585 (99%) | 62,829,476 (93%) | 572,374 (0.8%) |

[1] % of reads to align.
[2] Aligned reads filtered to remove duplicates and reads with mapping quality <10.
[3] % of filtered aligned reads.
[4] Properly paired: both the read and its mate are mapped to opposing strands of the reference sequence, with 3' ends innermost and 5' ends within the allowed distance from each other (0-500 bp).
