# Supplementary material for: An Africa-wide genomic evolution of insecticide resistance in the malaria vector Anopheles funestus involves selective sweeps, copy number variations, gene conversion and transposons
Source: PLoS Genet. 2020 Jun 4;16(6):e1008822. doi: 10.1371/journal.pgen.1008822 (PMC7297382; doi:10.1371/journal.pgen.1008822)
Supplement: S3 Table — (PDF) [file pgen.1008822.s010.pdf]

Sample ID	c5	c25	c50	c75	c95	Sites (coverage >10x and <=c95)	SNPs (coverage >10x and <=c95)
GHA-Obuasi-2014	19	28	33	38	46	177,211,238	3,673,050
BEN-Kpome-2015	24	36	41	47	56	176,871,101	4,537,744
CMR-Mebellom-2014	18	28	32	37	45	176,727,090	3,634,974
COD-Kinshasa-2015	18	28	33	38	46	176,871,745	4,033,839
COD-Mikalayi-2015	28	41	47	53	63	177,708,180	4,681,505
UGA-Tororo-2014	25	37	42	48	57	178,189,831	4,090,051
MWI-Chikwawa-2014	8	13	16	20	25	166,190,116	2,096,950
MWI-Chikwawa-2002	26	38	43	49	58	178,444,376	4,505,444
MOZ-Manhica-2016	25	38	44	49	59	178,817,543	4,248,748
MOZ-Morrumbene-2002	22	34	40	45	55	178,278,864	4,287,446
