# Supplementary material for: An Africa-wide genomic evolution of insecticide resistance in the malaria vector Anopheles funestus involves selective sweeps, copy number variations, gene conversion and transposons
Source: PLoS Genet. 2020 Jun 4;16(6):e1008822. doi: 10.1371/journal.pgen.1008822 (PMC7297382; doi:10.1371/journal.pgen.1008822)
Supplement: S4 Table — (PDF) [file pgen.1008822.s011.pdf]

Gene	Accession	Sequences	Location/Colony [1]	Reference
CYP6P9a	AFUN015792 (Vectorbase ID)	1	FUMOZ colony (MOZ)	Neafsey et al., 2015.
CYP6P9a	EU852647	1	FUMOZ colony (MOZ)	Wondji et al., 2009.
CYP6P9a	KR866038-KR866041	4	FANG colony (AGO)	Ibrahim et al., 2015.
CYP6P9a	KU168998-KU169011	14	GHA	Barnes et al., 2017b.
CYP6P9a	KR866042-KR866045	4	BEN	Ibrahim et al., 2015.
CYP6P9a	KU168962-KU168977	16	BEN	Barnes et al., 2017b.
CYP6P9a	KU168978-KU168997	20	CMR	Barnes et al., 2017b.
CYP6P9a	KR866034-KR866037	4	UGA	Ibrahim et al., 2015.
CYP6P9a	KU169012-KU169029	18	UGA	Barnes et al., 2017b.
CYP6P9a	KJ150655-KJ150664	10	ZMB	Riveron et al., 2014.
CYP6P9a	KR866026, KR866027, KR866030, KR866033	4	ZMB	Ibrahim et al., 2015.
CYP6P9a	JX627280-JX627289	10	MWI	Riveron et al., 2013.
CYP6P9a	KR866022, KR866023, KR866028, KR866032	4	MWI	Ibrahim et al., 2015.
CYP6P9a	KP984806-KP984865	60	MWI	Barnes et al., 2017a.
CYP6P9a	KU169030-KU169049, KU169072-KU169103	52	MWI	Barnes et al., 2017b.
CYP6P9a	JX627267-JX627279	13	MOZ	Riveron et al., 2013.
CYP6P9a	KR866024, KR866025, KR866029, KR866031	4	MOZ	Ibrahim et al., 2015.
CYP6P9a	KU169050-KU169071, KU169104-KU169123	42	MOZ	Barnes et al., 2017b.
CYP6P9b	AFUN015889 (Vectorbase ID)	1	FUMOZ colony (MOZ)	Neafsey et al., 2015
CYP6P9b	EU852648	1	FUMOZ colony (MOZ)	Wondji et al., 2009.
CYP6P9b	KR866062-KR866065	4	FANG colony (AGO)	Ibrahim et al., 2015.
CYP6P9b	KR866066-KR866069	4	BEN	Ibrahim et al., 2015.
CYP6P9b	KR866058-KR866061	4	UGA	Ibrahim et al., 2015.
CYP6P9b	KR866050, KR866051, KR866053, KR866054	4	ZMB	Ibrahim et al., 2015.
CYP6P9b	KJ150665-KJ150674	10	ZMB	Riveron et al., 2014.
CYP6P9b	KP984866-KP984925	60	MWI	Barnes et al., 2017a.
CYP6P9b	JX627303-JX627312	10	MWI	Riveron et al., 2013.
CYP6P9b	KR866046, KR866048, KR866052, KR866055	4	MWI	Ibrahim et al., 2015.
CYP6P9b	JX627290-JX627302	13	MOZ	Riveron et al., 2013.
CYP6P9b	KR866047, KR866049, KR866056, KR866057	4	MOZ	Ibrahim et al., 2015.

[1] Countries are represented by ISO 3166-1 alpha-3 codes: MOZ=Mozambique; AGO=Angola; GHA=Ghana; BEN=Benin; CMR=Cameroon; UGA=Uganda; ZMB=Zambia; MWI=Malawi.
