# Supplementary material for: An Africa-wide genomic evolution of insecticide resistance in the malaria vector Anopheles funestus involves selective sweeps, copy number variations, gene conversion and transposons
Source: PLoS Genet. 2020 Jun 4;16(6):e1008822. doi: 10.1371/journal.pgen.1008822 (PMC7297382; doi:10.1371/journal.pgen.1008822)
Supplement: S5 Table — (PDF) [file pgen.1008822.s012.pdf]

Country	Location	GPS Coordinates	Collection date	Climate
Mozambique	Palmeira	25°15’19’’S, 32°52’22’’E	2016	Tropical
Mozambique	Morrumbene	23°39’54’’S, 35°20’13’’E	2002	Tropical
Malawi	Chikwawa	16°1’ S, 34°47’ E	2014	Tropical
Malawi	Chikwawa	16°1’S, 34°47’E	2002	Tropical
Zambia	Kaoma	14°48’00”S, 24°48’00”E	2013	Tropical
DR Congo	Mikalayi	6°1’27”S, 22°19’5”E	2015	Equatorial
DR Congo	Kinshasa	4°19’39”S, 15°18’48”E	2015	Equatorial
Uganda	Tororo	0°45’N, 34°5’E	2014	Tropical/Equatorial
Cameroon	Mibellon	6°46′ N, 11°70′E	2015	Tropical/Equatorial
Benin	Kpome	6°23’N, 2°13’E	2015	Tropical/Equatorial
Ghana	Obuasi	5°56′N, 1°37′W	2014	Tropical/Equatorial
